# Pancancer Analysis and the Oncogenic Role of *UBTF* in Breast Invasive Carcinoma

**DOI:** 10.3390/ijms27062909

**Published:** 2026-03-23

**Authors:** Mingang He, Yi Wu, Simeng Liu, Yifeng Hou, Hefen Sun, Wei Jin

**Affiliations:** 1 Key Laboratory of Breast Cancer in Shanghai, Fudan University Shanghai Cancer Center, Shanghai 200032, China; 23111230006@m.fudan.edu.cn (M.H.);; 2 Department of Oncology, Shanghai Medica College, Fudan University, Shanghai 200032, China

**Keywords:** *UBTF*, pancancer, tumor microenvironment, immune response, biomarker

## Abstract

Upstream binding transcription factor (*UBTF*) is a nuclear transcription factor implicated in ribosome biogenesis, yet its pancancer relevance and immunological associations remain incompletely understood. We integrated datasets from The Cancer Genome Atlas (TCGA), Genotype-Tissue Expression (GTEx), Human Protein Atlas (HPA), Cancer Cell Line Encyclopedia (CCLE), and cBioPortal databases to characterize *UBTF* expression, genomic alterations, and prognostic value across 33 cancer types. Immune microenvironment analyses were performed using ESTIMATE and multiple deconvolution algorithms. CRISPR-Cas9–mediated *UBTF* depletion was conducted in breast invasive carcinoma (BRCA) cell lines to evaluate functional roles. *UBTF* was broadly upregulated in multiple tumors with recurrent copy number gains. Survival analyses revealed cancer type–dependent prognostic associations. *UBTF* expression correlated with immune/stromal contexture, checkpoint features, and predicted immunotherapy response. In BRCA, *UBTF* depletion reduced proliferation and migration while increasing apoptosis. A *UBTF*-related prognostic signature effectively stratified patient outcomes and was associated with immune infiltration and predicted immunotherapy response. *UBTF* represents a pancancer biomarker linked to tumor immunity, with validated functional significance in BRCA and potential utility for risk stratification.

## 1. Introduction

Upstream binding transcription factor (*UBTF*), which encodes the protein *UBTF*, is located on chromosome 17q21.31 and produces a 764–amino acid HMG-box DNA-binding protein [1]. *UBTF* is a core component of the RNA polymerase I (Pol I) preinitiation complex and facilitates rDNA transcription by promoting Pol I binding at rDNA promoter regions [1,2]. Beyond this canonical role, *UBTF* has also been reported to localize broadly to RNA polymerase II (Pol II)-transcribed genes across the human genome [3,4], suggesting that *UBTF* is widely involved in transcriptional regulation.

Consistent with its positioning at the nexus of biosynthesis and transcription, *UBTF* has been linked to cellular programs that are central to malignant behavior. Prior work has implicated *UBTF* in DNA damage and repair processes within ATR/ATM-regulated DNA damage responses, and in signaling responses to growth factor stimulation [4]. *UBTF* has also been connected to the regulation of differentiation, proliferation, and cell growth through multiple pathways [5], and its activity can be modulated through interactions with key repressors of cell-cycle progression, including RB family proteins and p53 [6]. These observations provide a biological rationale for examining *UBTF* in cancer, where increased biosynthetic demand and altered transcriptional states are common.

Although *UBTF* has not been extensively studied across tumor types, emerging evidence supports its relevance in cancer pathogenesis. *UBTF* upregulation has been reported in lung cancer specimens [7], and a recent study linked *UBTF* to tumor progression in melanoma [8]. In parallel, *UBTF* genetic alterations, including fusion events and recurrent internal tandem duplications reported in AML cohorts [9,10,11], are associated with hematologic malignancy disease subtype and clinical outcome [10,12]. Notably, recent work in AML further suggested that *UBTF* may contribute to immune escape through *PD-L1* regulation [13], suggesting that *UBTF* can intersect not only with tumor-intrinsic growth programs but also with tumor–immune interactions. These malignancies represent substantial global health burdens. According to the Global Cancer Observatory (GLOBOCAN) 2020 data, breast cancer is the most commonly diagnosed cancer worldwide, with 2.3 million new cases annually, lung cancer accounts for 2.2 million cases with the highest mortality rate, and melanoma represents approximately 325,000 new cases globally [14]. Collectively, these cancers contribute significantly to cancer-related disability-adjusted life years (DALYs), with breast cancer alone accounting for over 17 million DALYs globally [15]. The high prevalence and disease burden of these cancers underscore the importance of identifying novel biomarkers and therapeutic targets such as *UBTF*.

Moreover, cancer is being increasingly recognized as an ecosystem in which tumor cells and the tumor microenvironment coevolve [16,17]. Immune infiltration, stromal composition, and immune checkpoint activity can influence prognosis and the response to immune checkpoint blockade [18,19]. Importantly, the immune landscape is not solely a property of tissue context; it can be coupled to tumor genomic and transcriptional states [19]. Whether *UBTF* expression is systematically associated with these immune features across cancers—and how such associations are related to clinical outcomes—remains unclear.

Here, we performed an integrative pancancer analysis to characterize *UBTF* expression patterns and molecular alterations and to evaluate their associations with prognosis and immune contexture across 33 cancer types. We further focused on breast invasive carcinoma (BRCA) to connect *UBTF*-associated immune and pathway programs with functional evidence from CRISPR-Cas9–mediated *UBTF* depletion in BRCA cell lines, and we developed and externally validated a *UBTF*-related prognostic signature to support risk stratification and immune relevance in BRCA.

## 2. Results

### 2.1. Pancancer Expression Profile of UBTF

**Figure 1:**
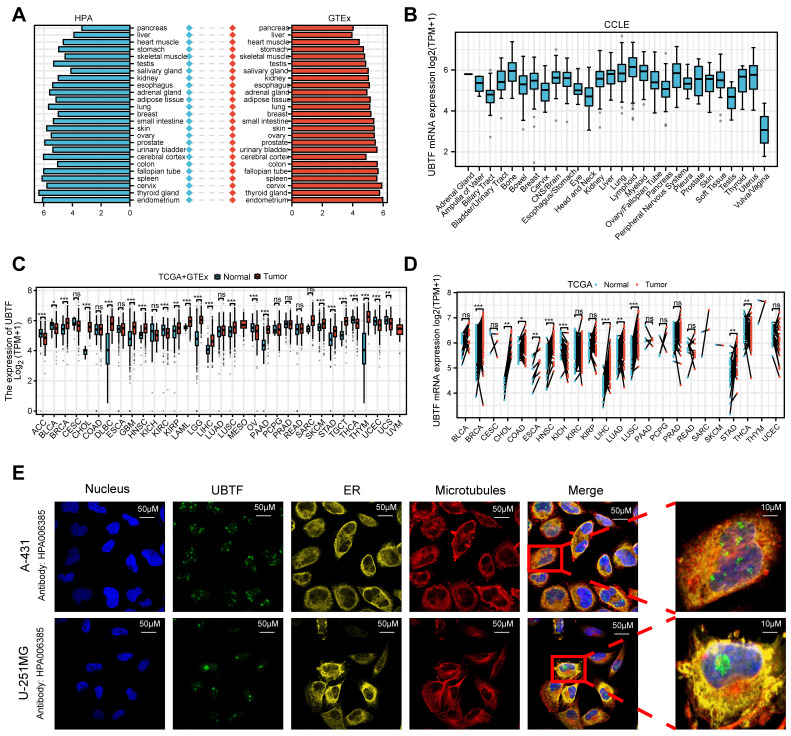
*UBTF* Expression Patterns Across Normal Tissues and Cancer Types. (**A**) Normal tissue *UBTF* expression profiles (HPA + GTEx datasets); (**B**) Cancer cell line *UBTF* expression profiles (Cancer Cell Line Encyclopedia (CCLE) datasets); (**C**) Comparative *UBTF* expression in normal versus malignant tissues (TCGA + GTEx datasets); (**D**) *UBTF* mRNA expression across tumor tissues compared with paired normal tissues (TCGA datasets); (**E**) IF visualization of UBTF subcellular compartmentalization across nucleus, ER, and microtubules in A-431 and U-251MG cell lines (HPA database). Blue: nucleus (DAPI); Green: UBTF; Yellow: endoplasmic reticulum (ER); Red: microtubules. Merge: overlay of all channels. Scale bar: 10 μm. Statistical comparison was performed using the Wilcoxon rank-sum test *, *p* < 0.05; **, *p* < 0.01; ***, *p* < 0.001; ns, not significant.

Analysis of datasets from the Human Protein Atlas (HPA) and the Genotype-Tissue Expression (GTEx) project revealed that *UBTF* mRNA expression was elevated in the endometrium, thyroid gland, spleen, and other normal tissues (Figure 1A). Analysis of the Tumor Immune Estimation Resource 2.0 (TIMER 2.0) dataset further revealed increased *UBTF* mRNA expression in multiple cancer types (Supplementary Figure S1A). Similarly, *UBTF* protein levels were elevated in various cancers (Supplementary Figure S1B,C), as supported by immunohistochemistry (IHC) data from the Human Protein Atlas (HPA) database (Supplementary Figure S1D–F). Furthermore, analysis of cancer cell lines revealed that *UBTF* is highly expressed in specific cancers, such as bone, pancreatic, and uterine cancers (Figure 1B). Combined analysis of the TCGA and GTEx datasets confirmed that *UBTF* is highly expressed in several cancers, including BRCA (Figure 1C). Receiver operating characteristic (ROC) analysis demonstrated the diagnostic value of *UBTF* (area under the curve (AUC) > 0.7) in BRCA and other cancers (Supplementary Figure S2). These findings were validated in paired tumor and normal tissue samples (Figure 1D). Finally, immunofluorescence (IF) staining from the HPA database indicated that the *UBTF* protein is located primarily in the nucleus (Figure 1E).

### 2.2. Genomic and Epigenetic Alterations of UBTF

*UBTF* genetic alterations spanning multiple malignancy types were interrogated via the cBioPortal database. *UBTF* was altered in 2% of pancancer samples, predominantly via missense mutations and amplification (Supplementary Figure S3A). *UBTF* alteration was associated with high-frequency coalteration of several genes, including ALOX12P1, THNS2_AST, and NUP210P1 (Supplementary Figure S3B). Missense mutations were the predominant variant type, and single-nucleotide variants (SNVs) showed a strong C > T bias (69%) (Supplementary Figure S3C). Copy-number variants (CNVs) of *UBTF* was significantly associated with poor prognosis in several cancers, including MESO, PRAD, LUSC, and COAD. *UBTF* was associated with frequent genetic alterations, with mutation rates exceeding 4% in PCPG, UCEC, and PRAD (Supplementary Figure S4A). Most mutations were missense, followed by truncating and splice-site variants, with a recurrent G601S missense mutation observed (Supplementary Figure S4B). Mutation burden varied by cancer type and was highest in UCEC and SKCM (Supplementary Figure S4C). *UBTF* mRNA expression correlated positively with copy number (Spearman’s ρ = 0.14, *p* < 0.05; Supplementary Figure S4D), though the modest coefficient indicates that genomic amplification explains only part of expression variability. *UBTF* expression increased with increasing copy number, peaking in the amplified samples (Supplementary Figure S4E).

Additionally, we obtained the details of the SNV and CNV of *UBTF* across multiple cancer types from the GSCA database. CNV of *UBTF* was significantly associated with poor prognosis in several cancers, including UCEC, LGG, ACC, and PRAD (Supplementary Figure S3D). CNV was positively correlated with mRNA expression across cancers, including BRCA, OV, KIRP, and LUAD (Supplementary Figure S3E). *UBTF* SNVs were associated with poor survival in patients with BRCA, UCEC, and COAD (Supplementary Figure S4F). The mutation frequency ranged from 1% to 16% across cancers, with UCEC having the highest mutation frequency (Supplementary Figure S4G).

Next, we evaluated the methylation levels of *UBTF* in BRCA using the UALCAN database [18,19], and we evaluated methylation sites using the MEXPRESS database [20,21]. In BRCA, *UBTF* promoter methylation was significantly greater in tumors than in normal tissues (*p* < 0.05) (Supplementary Figure S4H), indicating epigenetic regulation. The genomic context of *UBTF* was revealed to be within a predicted CpG island, with methylation at specific probes (e.g., cg04583165) significantly correlated with its expression (r = −0.083, FDR < 0.05) in BRCA (Supplementary Figure S3F).

### 2.3. Prognostic Value Across Cancer Types

**Figure 2:**
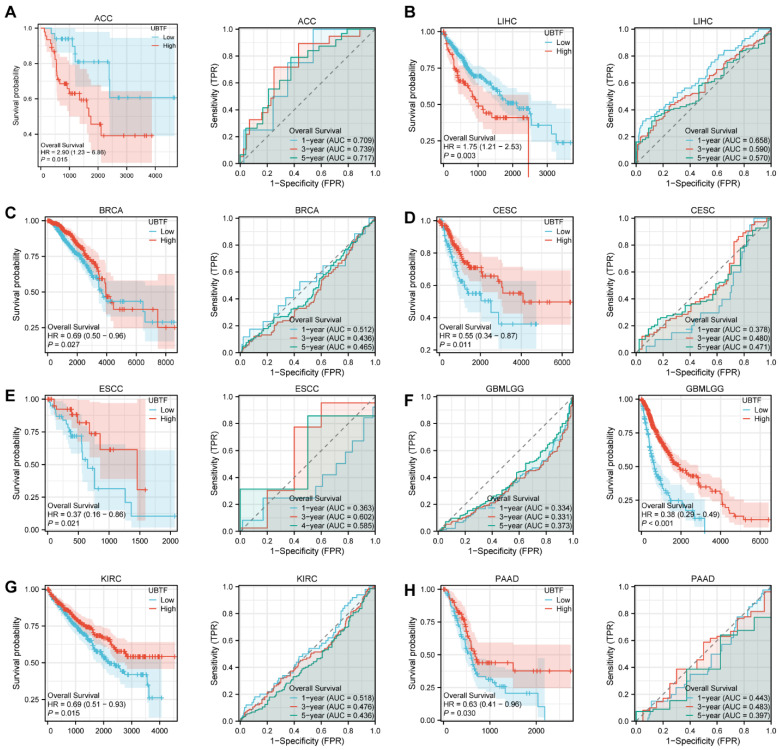
Prognostic Value of *UBTF* in Multiple Human Cancers. KM survival curves and ROC analysis between *UBTF* expression and OS of ACC (**A**), LIHC (**B**), BRCA (**C**), CESC (**D**), ESCC (**E**), GBMLGG (**F**), KIRC (**G**), PAAD (**H**). Log-rank test was used for survival analysis. Left panels: Kaplan–Meier survival curves comparing high (red) and low (blue) UBTF expression groups. Shaded areas represent 95% confidence intervals. Right panels: ROC curves for 1-year, 3-year, and 5-year overall survival prediction. Shaded areas indicate the area under the ROC curve (AUC).

TCGA RNA-seq and clinical data were analyzed to further elucidate *UBTF* abundance’s prognostic significance in malignancies. Kaplan–Meier (KM) survival curves were constructed and ROC analysis was executed to explore the association between *UBTF* abundance and overall survival (OS) in 33 malignancy categories. Elevated *UBTF* abundance was significantly associated with poor outcomes in patients with ACC and LIHC (Figure 2A,B). In contrast, elevated *UBTF* abundance levels were positively associated with better prognosis in BRCA, CESC, ESCC, GBMLGG, KIRC, and PAAD (Figure 2C–H). To exclude nontumor event bias, we further assessed *UBTF* abundance’s effect on disease-specific survival (DSS). Findings paralleled those from OS analysis, revealing that increased *UBTF* abundance predicted worse outcomes in patients with LIHC, ACC, and UVM (Supplemental Figure S5A–C). Moreover, *UBTF* abundance was inversely associated with DSS in BRCA, GBMLGG, and KIRC (Supplemental Figure S5D–F). Furthermore, we appraised the association between *UBTF* and progression-free interval (PFI). Poor prognosis was associated with elevated *UBTF* abundance in patients with ACC, ESAD, LIHC, LUSC, PRAD, STAD, SARC, and UVM (Supplemental Figure S6A–H). Moreover, *UBTF* abundance was inversely associated with DSS in ESCC, GBM, BRCA, CHCL, UCEC, and GBMLGG (Supplemental Figure S6I–N).

### 2.4. Clinicopathological and Immune Subtype Associations

Accurate tumor subtype classification is vital for prognostic evaluation and tailoring precision therapeutic strategies. This study evaluated how *UBTF* expression correlates with clinicopathological characteristics across diverse tumor types. We observed elevated *UBTF* expression in patients aged ≤50 years with ESCA, HNSC, KIRP, OSCC, COAD, READ, SARC, THYM, and GBMLGG but in patients aged >50 years with CESC (Supplemental Figure S7A–J). Moreover, *UBTF* expression increased with increasing tumor grade in HNSC, LIHC, KIRC, OSCC, and UCEC (Supplemental Figure S7K–O). In LIHC and ESCA, *UBTF* expression was higher in patients with a body mass index (BMI) > 25 (Supplemental Figure S7P,Q). *UBTF* expression tended to increase with increasing TNM stage in LIHC, LUADLUSC and SKCM (Supplemental Figure S7R–T). We also examined *UBTF* expression-immune subtype associations. *UBTF* exhibited distinct distribution patterns across immune subtype categories in BLCA, BRCA, CHOL, TGCT, KIRC, LUAD, STAD, LUSC, LIHC, PRAD, and THCA (Supplemental Figure S8).

### 2.5. Pancancer Immune Landscape and Immunotherapy Response Prediction

**Figure 3:**
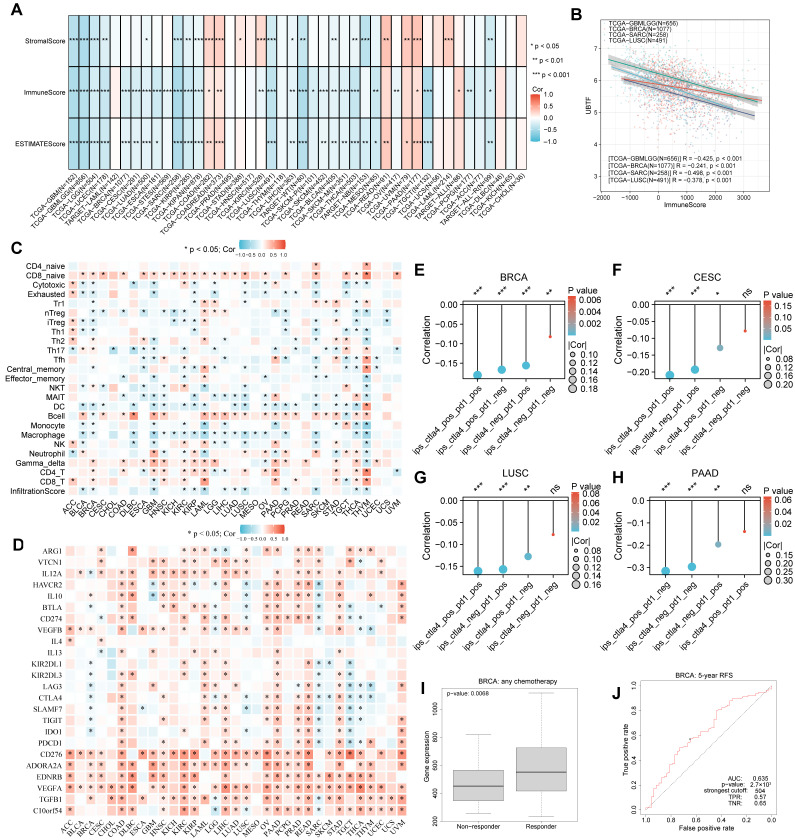
Pancancer Association of *UBTF* with Immune Microenvironment and Predicted Immunotherapy Response. (**A**) Correlations between *UBTF* expression and StromalScore, ImmuneScore and ESTIMATEScore. (**B**) Top 3 cancers with the highest immune score; (**C**) Correlation between *UBTF* expression and immune cell infiltration across cancer types (GSVA); (**D**) Correlation between *UBTF* expression and immune checkpoints across cancer types; (**E**–**H**) Correlation between *UBTF* and predicted immunotherapy response across cancer types; (**I**,**J**) Box plots showing the differences in *UBTF* expression between responders and nonresponders, and ROC curve showing the predictive accuracy of patient therapeutic response according to *UBTF* levels (ROCplotter database). Spearman correlation was used for correlation analysis. Wilcoxon rank-sum test was used for group comparisons. * *p* < 0.05; ** *p* < 0.01; *** *p* < 0.001. ns = not significant (*p* > 0.05).

To characterize the tumor immune microenvironment, we computed immune, stromal, and ESTIMATE scores. Across various malignancy types, *UBTF* abundance demonstrated inverse relationships with all three scoring metrics (Figure 3A). Specifically, we observed robust positive associations between *UBTF* and immune, stromal, and ESTIMATE scores in GBM, BRCA, SARC, and LUSC (Figure 3B). Multiple computational methods including single-sample gene set enrichment analysis (ssGSEA) (Figure 3C), EPIC, CIBERSORT, xCELL, MCP-counter, TIMER, and QUANTISEQ (Supplemental Figure S9A–F), were employed to appraise the association of *UBTF* with immune cell infiltration among various malignancies. The outcomes disclosed that *UBTF* abundance was inversely correlated with the abundance of multiple immune cells in BRCA, GBM, KIRP, and THYM. In addition, *UBTF* abundance was inversely correlated with the abundance of various immune checkpoint genes in BRCA, SARC, and TGCT (Figure 3D).

Furthermore, we appraised the influence of *UBTF* on predicted immunotherapy response across malignancy categories. Immunophenoscore (IPS) served to evaluate associations between *UBTF* levels and predicted immunotherapy outcomes, and the outcomes disclosed a robust inverse association between *UBTF* and predicted immunotherapy response to *PD-1*, *CTLA4*, and combination therapy in BRCA, CESC, LUSC, and PAAD (Figure 3E–H). We further explored *UBTF*’s capacity to predict therapeutic response in malignancies through the ROC plotter database, which indicated that Chemotherapy-responsive BRCA patients demonstrated elevated *UBTF* levels, achieving an AUC value of 0.635 for 5-year recurrence-free survival (RFS) (Figure 3I,J).

### 2.6. Functional Characterization in BRCA

**Figure 4:**
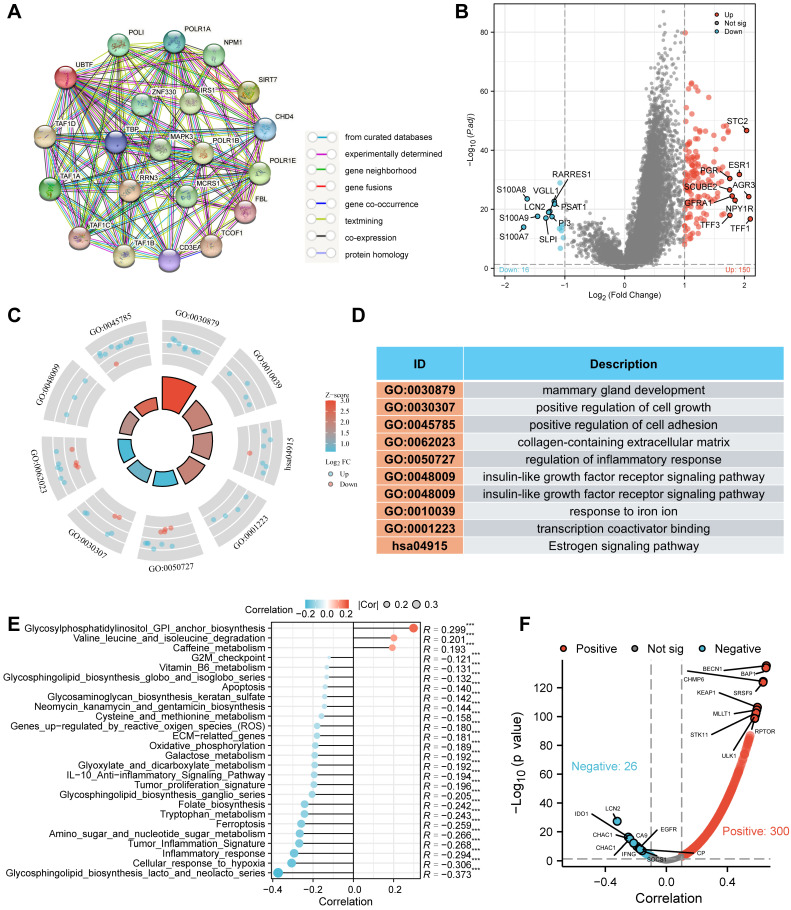
Functional Characterization of *UBTF* in Breast Cancer. (**A**) *UBTF*-centered protein interaction network; (**B**) Volcano visualization of gene expression differences between *UBTF*-high and *UBTF*-low cohorts (FDR-adjusted *p* < 0.05, |log_2_ fold-change (FC)| > 1); (**C**,**D**) GO and KEGG functional enrichment analysis; (**E**) Association between *UBTF* and pathways in BRCA (ssGSEA); (**F**) Association between *UBTF* and ferroptosis-related genes in BRCA. Spearman correlation was employed for pathway and gene correlation analysis (**E**,**F**). Dashed lines indicate significance thresholds: in (**B**), vertical dashed lines represent |log_2_(FC)| = 1 and horizontal dashed line represents *p* = 0.05; in (**E**), vertical dashed lines represent |R| = 0.1 and horizontal dashed line represents *p* = 0.05. *** *p* < 0.001.

We further investigated *UBTF*’s functional roles in BRCA by analyzing protein interaction networks and gene expression profiles. STRING database served as the source for *UBTF* protein interaction data (Figure 4A). In BRCA, differential gene expression profiling unveiled 150 genes with elevated expression and 116 genes with reduced expression (Figure 4B). Functional annotation via Gene Ontology (GO) revealed predominant DEG enrichment in biological processes including positive regulation of cell adhesion and growth, plus iron ion response; conversely, Kyoto Encyclopedia of Genes and Genomes (KEGG) pathway analysis identified estrogen signaling pathway as the principal enriched route (Figure 4C,D). ssGSEA disclosed that *UBTF* abundance was significantly inversely correlated with cellular response to hypoxia, inflammatory response, ferroptosis, tumor proliferation, extracellular matrix (ECM)-related genes, and apoptosis (Figure 4E). Because the apoptosis gene set comprises genes promoting and inhibiting apoptosis, we evaluated how each apoptosis-related gene correlates with *UBTF*. Multiple antiapoptotic factors exhibited significant inverse associations with *UBTF* abundance, including GAL, CTSC, and caspase-5, and significantly positively correlated with the abundance of proapoptotic factors, such as *ZNF830*, *BECN1*, *AMBRA1*, and *RGL2* (Supplementary Figure S10C). The pronounced association between *UBTF* and ferroptosis prompted us to investigate how *UBTF* correlates with 484 ferroptosis-associated genes catalogued in the FerrDB database. Analysis identified 326 significantly differentially expressed genes, including 26 that exhibited significant negative correlation with *UBTF* (Figure 4F). We further explored *UBTF* through coessentiality analysis, which uncovered 35 functionally related neighboring genes (Supplementary Figure S10A), which were enriched predominantly in ribosome and cadherin binding (Supplementary Figure S10B).

### 2.7. Immune Characteristics in BRCA

**Figure 5:**
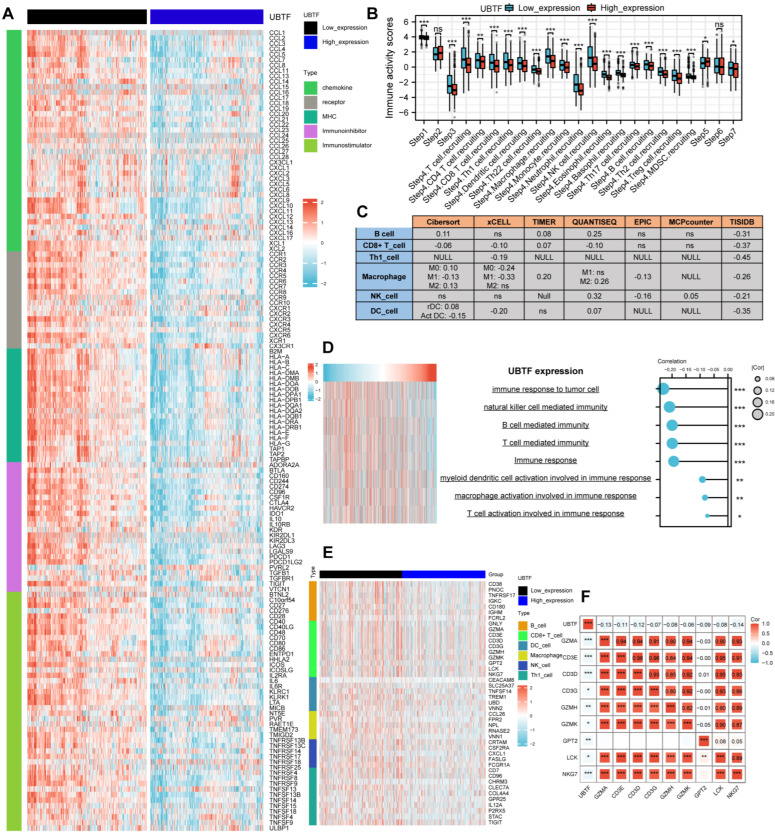
Immune Landscape of *UBTF* in BRCA. (**A**) Associations between *UBTF* and immune modulators in BRCA; (**B**) Cancer immune cycle step differences between high- and low-*UBTF* cohorts; (**C**) Seven algorithms assess *UBTF*-immune cell infiltration associations in BRCA; (**D**) GSVA assesses *UBTF*-immune pathway associations; (**E**) Effector gene differences in tumor-associated immune cells between high- and low-*UBTF* groups; (**F**) Links between *UBTF* expression and *CD8* + T-cell marker gene expression in BRCA. Spearman correlation was employed for correlation analysis. Wilcoxon rank-sum test was employed for group comparisons. * *p* < 0.05; ** *p* < 0.01; *** *p* < 0.001. ns = not significant (*p* > 0.05).

*UBTF* expression showed strong negative correlations with multiple immune modulators in BRCA (Figure 5A). The *UBTF* low-expression group demonstrated stronger anticancer immune activity across multiple steps of the cancer-immunity cycle, including priming and activation (step 3), trafficking of immune cells to tumors (step 4), immune cell infiltration into tumors (step 5), and killing of cancer cells (step 7) (Figure 5B). Immune cell infiltration analysis using multiple algorithms consistently revealed negative correlations between *UBTF* expression and infiltration of *CD8*+ T cells, NK cells, and macrophages (Figure 5C). Significant negative associations were observed between *UBTF* expression and immune-related pathways encompassing cytokine production, T-cell-mediated immunity, and tumor-targeting immune response (Figure 5D). Analysis of immune cell marker gene expression confirmed significant negative correlations between *UBTF* and marker genes of infiltrating immune cells (Figure 5E), particularly macrophage markers *CD11B* and *CD45* (Figure 5F).

### 2.8. Experimental Validation of UBTF Function in BRCA

**Figure 6:**
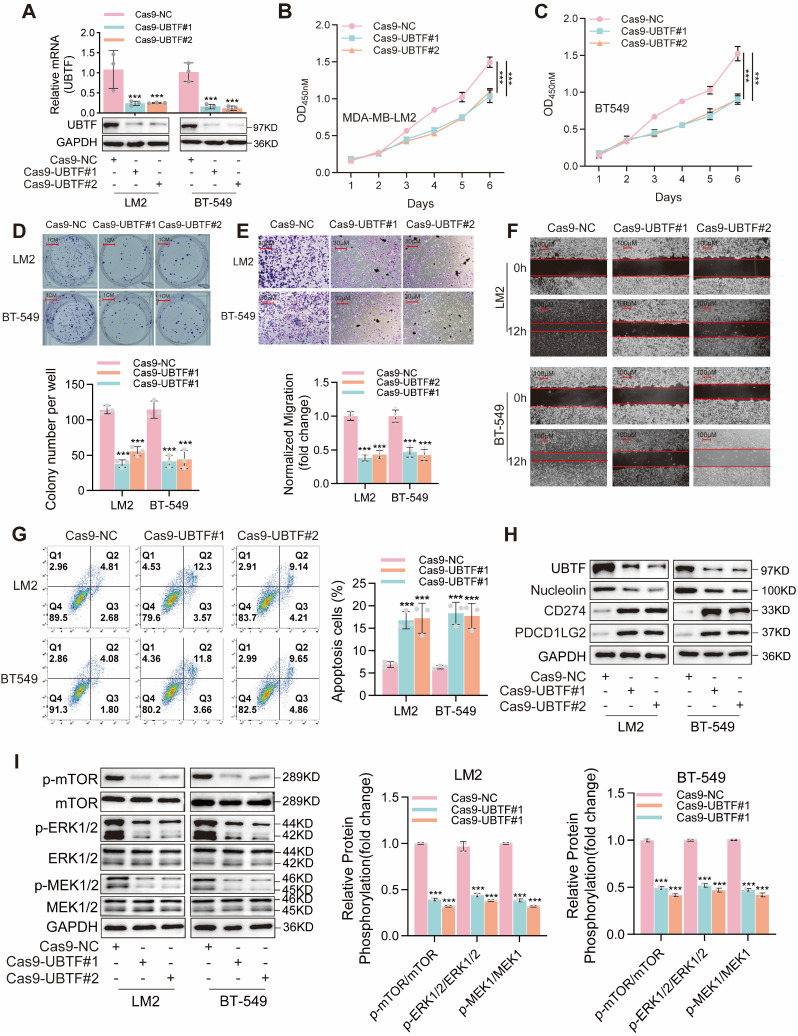
*UBTF* Knockdown Inhibits Malignant Phenotypes and Increases Immune Checkpoint Expression in BRCA Cells. (**A**) *UBTF* mRNA and protein expression in LM2 and BT-549 cells after CRISPR-Cas9 knockdown. (**B**,**C**) Cell viability measured by CCK-8 assay over 6 days. (**D**) Colony formation assay showing representative images and quantification. Colonies counted in a blinded manner. Scale bar: 1 cm. (**E**) Transwell migration assay showing representative images and quantification. (**F**) Wound healing assay at 0 h and 12 h. Red lines indicate wound edges. Scale bar: 100 μm. (**G**) Flow cytometry analysis of apoptosis. Q1: necrotic; Q2: late apoptotic; Q3: viable; Q4: early apoptotic cells. Flow cytometry dot plots showing cell distribution. Colors represent cell density: blue indicates low cell density, green/yellow indicates medium density, and red indicates high density. (**H**) Western blot analysis of *Nucleolin*, *PD-L1*, and *PD-L2* expression. (**I**) Western blot analysis of *mTOR/ERK/MEK* pathway activation. Quantification shows phosphorylated/total protein ratios normalized to Cas9-NC control. All experiments were performed in triplicate in three independent experimental sets. Data represent mean ± SD. Statistical analyses: two-way ANOVA with Dunnett’s post hoc test (**B**,**C**); one-way ANOVA with Dunnett’s post hoc test (**A**,**D**,**E**,**G**,**I**). *** *p* < 0.001. Original Western blot images are provided in Supplementary Figures S13 and S14.

To further validate the role of *UBTF* in BRCA cells, we knocked down the expression of *UBTF* in MDA-MB-231.LM2 (LM2) and BT-549 BRCA cells using CRISPR-Cas9. Quantitative real-time PCR (qRT-PCR) and Western blot analysis confirmed successful *UBTF* knockdown, with both mRNA and protein expression levels significantly reduced compared with the control group (Figure 6A). The ability of *UBTF* to affect the proliferation of BRCA cells was confirmed by a Cell Counting Kit-8 (CCK-8) assay (Figure 6B,C) and a colony formation assay (Figure 6D), which revealed that compared with the control group, *UBTF* knockdown markedly reduced the proliferation ability of LM2 and BT-549 cells. Additionally, the migration abilities of the LM2 and BT-549 cells significantly decreased after *UBTF* knockdown, as revealed by the results of the Transwell (Figure 6E) and wound healing (Figure 6F) assays, respectively. Flow cytometry analysis revealed that *UBTF* knockdown significantly increased the rate of baseline apoptosis in LM2 and BT-549 cells (Figure 6G).

To further investigate the molecular mechanisms, we examined key pathway components and immune-related molecules. Western blot analysis revealed that *UBTF* knockdown reduced the expression of Nucleolin, a ribosome biogenesis-related protein, and increased the levels of immune checkpoint proteins *PD-L1 (CD274)* and *PD-L2 (PDCD1LG2)* (Figure 6H). Additionally, *UBTF* knockdown significantly reduced the phosphorylation of *mTOR*, *ERK*, and *MEK*, indicating suppression of the *mTOR/ERK/MEK* signaling pathway (Figure 6I).

### 2.9. Development and Validation of UBTF-PS Prognostic Model

**Figure 7:**
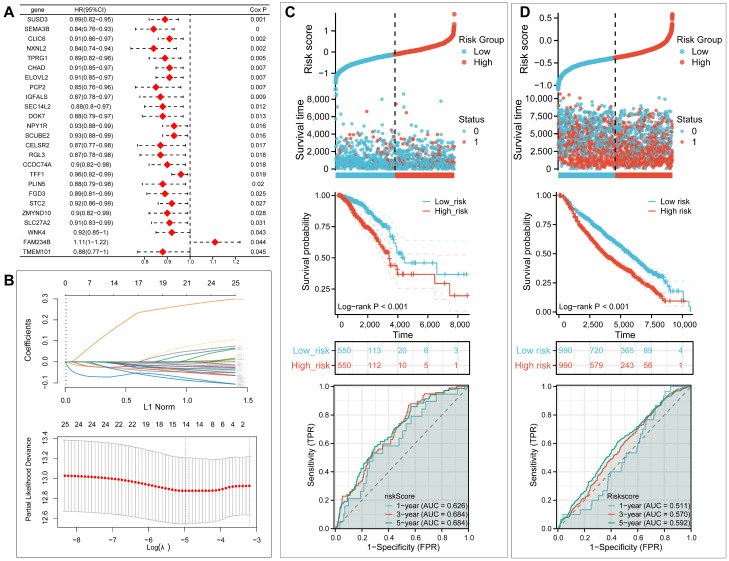
Development and Validation of *UBTF*-Related Prognostic Signature. (**A**) Forest plot of univariate Cox regression analysis for 14 *UBTF*-related genes in BRCA. Red = hazard ratios, black lines = 95% CI. (**B**) LASSO Cox regression analysis. Upper: coefficient trajectories; Lower: cross-validation for optimal λ selection. (**C**,**D**) Risk score distribution, survival status, Kaplan–Meier curves, and time-dependent ROC curves for TCGA-BRCA (**C**) and METABRIC (**D**) cohorts. Log-rank test was used for survival analysis. Shaded area in ROC curves represents the AUC.

Univariate Cox regression of DEGs was performed to assess elevated *UBTF* expression’s prognostic significance in BRCA tumor cells; 14 genes were selected for Least Absolute Shrinkage and Selection Operator (LASSO) Cox regression, from which a prognostic model was built using the lambda value corresponding to minimal cross-validated deviance (Figure 7A,B). For external validation, the Molecular Taxonomy of Breast Cancer International Consortium (METABRIC) cohort was stratified using the median risk score derived from TCGA. Analysis across both databases revealed that overexpression of these 14 genes was associated with markedly unfavorable patient outcomes (Figure 7C,D). To assess the incremental clinical utility of *UBTF*-PS, decision curve analysis (DCA) was performed comparing four models: *UBTF*-PS alone (Risk Score), age alone, TNM stage alone, and the combined model (Risk Score + Age + TNM Stage). DCA demonstrated that the combined model incorporating *UBTF*-PS provided superior net benefit compared to clinical variables alone across a wide range of threshold probabilities for predicting 1-year, 3-year, and 5-year survival (Supplementary Figure S11A–C), confirming that *UBTF*-PS adds incremental clinical value beyond standard prognostic factors.

To integrate clinical features into a comprehensive evaluation of patient prognosis, univariate Cox regression analysis was first performed including age, race (Asian/non-Asian), TNM stage (I-II/III-IV), and risk score (Supplementary Figure S11D). Variables with *p* < 0.05 (age, TNM stage, and risk score) were subsequently included in multivariable Cox regression analysis, revealing that all three factors were independent risk factors affecting survival outcomes in BRCA patients (Supplementary Figure S11E). On the basis of the point values assigned to each independent risk factor, a nomogram was constructed to provide an intuitive representation of individualized survival probability (Supplementary Figure S11F). Strong predictive performance was demonstrated by the nomogram, with AUC values reaching 0.807, 0.776, and 0.736 at 1-, 3-, and 5-year time points, respectively (Supplementary Figure S11G). The calibration plot graphically evaluated the accuracy of the nomogram-predicted probabilities, with data points close to the ideal line confirming high predictive reliability at 1-year, 3-year, and 5-year intervals (Supplementary Figure S11H). Our model’s robust prognostic performance and clinical applicability are confirmed by these results.

### 2.10. UBTF-PS Model: Immune Infiltration and Immunotherapy Response

Analysis of immune cell infiltration through CIBERSORT indicated that elevated risk scores were associated with increased levels of most immune cell populations (Supplementary Figure S12A). Correlations between risk scores and immune checkpoint gene expression were subsequently evaluated. The majority of immune checkpoint genes demonstrated significant upregulation in high-risk patients (Supplementary Figure S12B). A substantial number of components in the immune response cycle, such as the release of cancer cell antigens (Step 1), cancer antigen presentation (Step 2), and their transport to the tumor site (Step 4), including the recruitment of CD4+ T cells, dendritic cells, Th17 cells, and regulatory T cells (Tregs), were notably positively correlated with the risk score (Supplementary Figure S12C). We corroborated these result`s by applying the TIDE algorithm to estimate immunotherapy response rates in low-risk versus high-risk patient populations. Predicted immunotherapy response rates were significantly lower in the high-risk group versus the low-risk group (Supplementary Figure S12D).

## 3. Discussion

*UBTF* is a nucleolar transcription factor that regulates rDNA transcription and ribosome biogenesis [1,2,13]. Although *UBTF* has been implicated in several cancer types [3,8,20], its pancancer expression patterns, regulatory mechanisms, and clinical significance remain poorly characterized. In this study, we performed a comprehensive pancancer analysis of *UBTF* across 33 cancer types and validated its functional role in BRCA.

*UBTF* expression was elevated in multiple tumor types compared with normal tissues. This reflects the increased ribosome biogenesis demands of cancer cells [2,13]. Elevated ribosome biogenesis is a characteristic feature of cancer that supports high translational capacity and accelerated proliferation [21,22]. Pancancer analyses show that nucleolar proteins and RNA polymerase I components are frequently upregulated and represent promising therapeutic targets [23]. As a core component of the RNA polymerase I preinitiation complex, *UBTF* is functionally linked to oncogenic pathways including *mTOR*, *MYC*, and *MAPK/ERK* [22].

We investigated the mechanisms driving *UBTF* overexpression in cancer. Genomic alterations, including copy number variations and mutations, are known to directly influence gene expression levels and represent key drivers of oncogene activation in cancer [24]. At the genomic level, *UBTF* alterations occurred in approximately 2% of samples, mainly copy number gains. While *UBTF* expression showed a statistically significant positive correlation with copy number (Spearman’s ρ = 0.14, *p* < 0.05), the weak correlation coefficient suggests that copy number alterations are not the primary driver of *UBTF* overexpression, and other regulatory mechanisms likely play more substantial roles. Given that epigenetic dysregulation represents a key mechanism of gene expression control in cancer [25], we examined DNA methylation patterns in BRCA. Differential methylation was observed between tumors and normal tissues. While overall promoter methylation levels differed, specific CpG sites (e.g., cg04583165) showed inverse correlations with *UBTF* expression, suggesting site-specific regulatory effects. These observations indicate that *UBTF* expression in cancer is controlled by multiple mechanisms, including genomic alterations and epigenetic modifications.

The prognostic value of *UBTF* differed greatly by cancer type. High *UBTF* expression was associated with poor survival in ACC (HR = 2.31, *p* < 0.01) and LIHC (HR = 1.68, *p* < 0.01) but favorable outcomes in BRCA (HR = 0.71, *p* < 0.001), CESC, ESCC, GBMLGG, KIRC, and PAAD. This cancer type-dependent pattern is common in pancancer biomarker studies. Tumor purity, immune infiltration, and molecular subtypes vary substantially across cancer types [19], contributing to heterogeneous biomarker-outcome relationships. However, such prognostic heterogeneity likely reflects genuine biological differences in how *UBTF* functions across tumor contexts rather than technical artifacts. Similar cancer-specific patterns have been reported for stemness signatures in relation to immunotherapy response [22], supporting the concept that biomarker functions are context-dependent. These observations motivated us to validate *UBTF*’s functional role in a specific cancer type.

*UBTF* expression correlated with tumor immune microenvironment features across multiple cancer types. Seven algorithms showed consistent relationships between *UBTF* and immune/stromal scores (ssGSEA, CIBERSORT, TIMER, EPIC, xCell, MCP-counter, quanTIseq). In BRCA, GBM, KIRP, and THYM, higher *UBTF* expression was associated with reduced cytotoxic lymphocyte and antigen-presenting cell infiltration, consistent with an immunologically “cold” phenotype. *UBTF* also correlated with immune checkpoint expression (*PD-1*, *PD-L1*, *CTLA-4*, *LAG3*) in cancer-specific patterns. Immunophenoscore analyses suggested associations with predicted response to *PD-1/CTLA-4* blockade in BRCA, CESC, LUSC, and PAAD. These findings indicate that *UBTF* may influence tumor–immune interactions beyond its canonical role in ribosome biogenesis. Emerging evidence suggests that metabolic programs, including ribosome biogenesis, can modulate immune cell function and contribute to immunosuppressive microenvironments [26,27]. These observations suggest that *UBTF* connects ribosome biogenesis to immune modulation through metabolic and translational mechanisms.

Given the cancer-specific patterns observed in our pancancer analyses, we selected BRCA for functional validation based on its clinical importance, biological heterogeneity, and availability of large validation cohorts. Functional enrichment analyses of *UBTF* revealed significant associations with cell proliferation, migration, and oncogenic signaling pathways. To validate these associations, CRISPR-Cas9 gene editing has emerged as a powerful and precise tool for functional genomics studies in cancer research [28]. We therefore employed CRISPR-Cas9 to generate stable *UBTF* knockdown in LM2 and BT-549 BRCA cell lines. *UBTF* knockdown reduced proliferation and migration and increased apoptosis, confirming that *UBTF* contributes to malignant behavior in BRCA. At the molecular level, *UBTF* knockdown suppressed *mTOR/ERK/MEK* phosphorylation. These pathways play central roles in breast cancer progression [29]. *UBTF* knockdown also reduced Nucleolin expression, a marker of ribosome biogenesis activity. These findings confirm that *UBTF* links ribosome biogenesis to oncogenic signaling in BRCA. This is consistent with the established role of ribosome biogenesis in promoting tumor growth and survival [30]. These results support an oncogenic role for *UBTF* in breast cancer.

In BRCA, higher *UBTF* expression was associated with reduced immune cell infiltration and an immunologically “cold” phenotype. Additionally, *UBTF* expression showed negative correlations with immune checkpoint molecules. To validate this correlation, we examined *PD-L1* and *PD-L2* protein levels in *UBTF*-knockdown BRCA cells. Consistent with the negative correlation, *UBTF* knockdown increased *PD-L1* and *PD-L2* expression. These findings support a model in which *UBTF* promotes malignant behavior through oncogenic signaling pathways while simultaneously suppressing immune checkpoint expression. This pattern contrasts with recent findings in AML, where *UBTF* promotes immune escape through *PD-L1* upregulation [13], suggesting differential regulation of immune checkpoint expression between solid tumors and hematologic malignancies. Such cancer-specific variation in immune checkpoint regulation is increasingly recognized, with transcriptomic analyses demonstrating substantial heterogeneity in checkpoint expression patterns both between and within cancer types [31]. Collectively, these findings indicate that *UBTF* exerts distinct immunoregulatory effects in different tumor types.

Several limitations should be noted. Our analysis uses bulk transcriptomic data, which cannot resolve cell-type-specific effects. The immunotherapy predictions are based on computational algorithms and require validation in prospective cohorts. Mechanistic links between *UBTF* and immune features remain correlative.

Future studies should address several questions. First, whether *UBTF* expression can predict clinical benefit from immunotherapy requires validation in prospective cohorts. Second, *UBTF* may represent a therapeutic target via RNA polymerase I inhibitors such as CX-5461 [31]. Third, the interplay between epigenetic therapies that reshape the tumor immune microenvironment and *UBTF* regulation requires further investigation [32]. Advanced imaging technologies, including nanoscale imaging [33,34] and Z-stack confocal microscopy, combined with single-cell multi-omics profiling, could reveal *UBTF*’s binding dynamics and cell-type-specific functions.

## 4. Materials and Methods

### 4.1. Data Collection and Preprocessing

For 33 malignancy categories, we obtained transcriptomic profiles together with matching clinical metadata via the UCSC Xena platform [35], a repository furnishing standardized, batch-normalized data originating from TCGA and GTEx initiatives. The UCSC Xena computational framework employs a uniform bioinformatics protocol encompassing read alignment, gene-level quantification, and log2 (TPM + 1) transformation across TCGA malignant specimens and GTEx physiological tissue specimens, thereby attenuating systematic discrepancies between data repositories and facilitating integrative cross-platform investigations. Protein-level expression profiles, IHC images, and IF confocal microscopy images were sourced from the HPA (http://v13.proteinatlas.org/) (accessed on 10 June 2025) [36] to corroborate *UBTF* expression patterns and subcellular distribution. Gene abundance matrices for malignancy-derived cell lines were procured from the CCLE (http://www.broadinstitute.org/ccle/home) (accessed on 15 June 2025) [37]. Disease nomenclature adheres to TCGA classification standards. Comprehensive nomenclature details and specimen characteristics are delineated in Supplementary Table S1. For independent validation, the METABRIC dataset (https://www.cbioportal.org/study/summary?id=brca_metabric#summary) (accessed on 15 June 2025) [38] served as an external cohort to evaluate the prognostic robustness of the model in BRCA.

### 4.2. Pancancer Expression Profiling of UBTF

*UBTF* mRNA abundance in physiological human tissues was evaluated by synthesizing transcriptomic data from HPA and GTEx repositories. *UBTF* mRNA abundance across diverse malignancy categories was interrogated via TIMER 2.0 (https://compbio.cn/timer2/) (accessed on 18 June 2025) [39]. *UBTF* protein abundance was ascertained utilizing data from the Clinical Proteomic Tumor Analysis Consortium (CPTAC) (https://proteomics.cancer.gov/programs/cptac) (accessed on 23 June 2025) [40], while IHC staining patterns were retrieved from HPA to corroborate expression at the proteomic level. Subcellular distribution of *UBTF* was interrogated using IF images from HPA. *UBTF* abundance across malignancy cell lines was interrogated using CCLE. By harmonizing gene expression profiles from TCGA and GTEx, differential abundance of *UBTF* between malignant and physiological tissues was systematically interrogated across 33 malignancy categories, with subsequent validation in paired tumor-normal tissue specimens. ROC curves were constructed to appraise the discriminatory capacity of *UBTF* abundance in distinguishing malignant from physiological tissues.

### 4.3. Prognostic Value Assessment of UBTF

Malignant specimens from 33 cancer categories were dichotomized into high- and low-abundance cohorts predicated on the median *UBTF* abundance value, as this methodology ensures balanced cohort sizes for robust statistical comparison and circumvents bias from outlier values [41]. Survival outcomes between cohorts were contrasted using KM analysis implemented in the R package survival [42] (v3.2.10). Statistical significance was ascertained via Log-rank tests, with hazard ratios (HRs) alongside 95% confidence intervals (CIs) derived through univariate Cox proportional hazards regression. The association between *UBTF* abundance and three survival endpoints was interrogated: DSS, OS, and PFI. To appraise the prognostic discriminatory capacity of *UBTF* abundance, time-dependent ROC analysis was executed via the timeROC R package [43] (v0.4), with AUC values computed at 1-, 3-, and 5-year time points to quantify predictive performance.

### 4.4. Genomic and Epigenetic Alteration Analysis

The mutational landscape of *UBTF* across diverse malignancies was systematically interrogated using cBioPortal (http://www.cbioportal.org) (accessed on 27 June 2025) [44] predicated on the TCGA Pancancer Atlas Studies dataset, wherein mutation sites, types, and frequencies were extracted from the “Cancer Types Summary” and “Mutations” modules. Concurrently, CNVs coupled with SNVs data were retrieved and interrogated through GSCALite (http://bioinfo.life.hust.edu.cn/web/GSCALite/) (accessed on 10 July 2025) [45]. Potential associations were appraised through Spearman’s rank correlation analysis, which examined the linkage between CNV mutation proportions and *UBTF* mRNA abundance. Furthermore, the SMART database (http://www.bioinfo-zs.com/smartapp/) (accessed on 15 July 2025) [46] was employed to interrogate the association between *UBTF* and its methylation status; differential methylation analysis was executed to contrast methylation levels at individual sites between physiological and malignant tissues, and the association between *UBTF* abundance and 14 specific methylation sites was interrogated.

### 4.5. Clinicopathological Feature Correlation Analysis

To appraise the associations between *UBTF* abundance and clinicopathological characteristics, we executed comprehensive correlation analyses across all 33 malignancy categories using TCGA data. Clinical variables including age, tumor grade, BMI, disease stage, and immune subtypes were systematically interrogated for each malignancy category. Clinical variables were categorized as appropriate.

### 4.6. Pancancer Immune Landscape Analysis

To thoroughly investigate *UBTF* abundance in relation to the tumor immune microenvironment across malignancies, we appraised multiple immune-related parameters, including immune scores, immune cell infiltration levels, immunomodulators, and tumor immunophenotypic features. Stromal, immune, and ESTIMATE scores were computed for each malignant specimen predicated on *UBTF* abundance levels using the R package ESTIMATE (v1.0.13), which infers non-malignant cell composition from gene expression profiles [47]. Seven computational algorithms (ssGSEA, CIBERSORT, TIMER, EPIC, MCP-counter, xCell, and quanTIseq) were used to explore the *UBTF*-immune cell infiltration relationship across pancancer categories. Specifically, six algorithms (CIBERSORT, TIMER, EPIC, MCP-counter, xCell, and quanTIseq) were implemented using the immunedeconv R package (v2.0.3), which integrates multiple immune cell quantification methods [48]; CIBERSORT was executed with 1000 permutations, and only specimens with *p* < 0.05 were included; ssGSEA was executed using the GSVA R package (v1.48.3) [49] with default parameters. All other algorithms were executed with default settings as implemented in their respective methods.

### 4.7. Immunotherapy Response Prediction Analysis

To characterize *UBTF* abundance in relation to predicted immunotherapy response, IPS data originated from The Cancer Immunome Atlas (TCIA) (https://tcia.at/home) (accessed on 18 July 2025) [50], and correlations between *UBTF* expression and IPS for anti–*CTLA-4*, anti–*PD-1*, and combination therapy were assessed using Spearman correlation analysis. Additionally, to predict cancer therapy response, we utilized the ROC plotter database (http://www.rocplot.org/) (accessed on 20 July 2025) [51], which integrates gene expression and clinical outcome data to assess the predictive value of *UBTF* for treatment response. The predictive performance of *UBTF* expression for chemotherapy response in BRCA was evaluated, and AUC values for RFS were calculated.

### 4.8. BRCA-Specific Immune Characterization Analysis

For BRCA-specific immune characterization, we executed additional analyses predicated on Charoentong et al.’s study [50]. In BRCA, 122 immune modulators were identified and subsequently analyzed for their correlations with *UBTF* mRNA abundance. To appraise the anti-tumor immune status, we employed the TIP database (http://biocc.hrbmu.edu.cn/TIP/) (accessed on 29 July 2025) [52]. In BRCA, immune cell infiltration levels were further corroborated using TISIDB database (http://cis.hku.hk/TISIDB/browse.php) (accessed on 2 August 2025) [53]. Gene sets pertaining to specific immune processes were sourced from the AmiGO 2 portal (https://amigo.geneontology.org/amigo) (accessed on 4 August 2025), and associations with *UBTF* were ascertained via the GSVA R package [49] (v1.48.3). Furthermore, we interrogated the association between *UBTF* and established immune cell marker genes in BRCA. For the *UBTF*-Related Prognostic Signature (*UBTF*-PS) prognostic model, the TIDE algorithm was additionally utilized to evaluate predicted immunotherapy response, with subsequent comparison of TIDE scores between high- and low-risk cohorts.

### 4.9. Functional Enrichment and Pathway Analysis

Protein–protein interaction networks were prognosticated via the STRING database (https://string-db.org/) (accessed on 11 August 2025) [54]. To ascertain differentially expressed genes (DEGs) distinguishing *UBTF* high- from low-abundance cohorts in BRCA, we employed Limma R package [55] (v3.56.2), establishing significance thresholds at FDR-adjusted *p* value < 0.05 alongside |log_2_ FC| > 1. Subsequently, the identified DEGs underwent GO and KEGG pathway enrichment analyses via ClusterProfiler R package [56] (v4.1.4) with default parameters, wherein significantly enriched terms were ascertained at *p* value < 0.05.

To interrogate the association between *UBTF* and tumor-related pathway activities in BRCA, we collected gene sets from various biological pathways predicated on a previous study [57]. We executed ssGSEA to compute enrichment scores for each pathway across all BRCA specimens. The association between *UBTF* abundance and pathway enrichment scores was then appraised using Spearman correlation analysis. To interrogate the association between *UBTF* and ferroptosis in depth, the FerrDB database yielded 484 ferroptosis-related genes for analysis and interrogated their association with *UBTF* abundance in BRCA using Spearman correlation analysis, with significantly correlated genes defined as those with |R| > 0.1 and *p* < 0.05. Additionally, to identify genes functionally related to *UBTF*, we executed coessentiality analysis using DepMap database (https://depmap.org/portal/) (accessed on 12 August 2025), which identifies genes whose essentiality patterns correlate across hundreds of malignant cell lines [58].

### 4.10. Construction and Validation of UBTF-PS

Within the TCGA-BRCA cohort, patients were bifurcated into high- and low-*UBTF* abundance cohorts predicated on median abundance. These DEGs were then analyzed via univariate Cox regression for identifying OS-related genes. The glmnet R package [59] (v4.1-7) was utilized to build a *UBTF*-related prognostic model via LASSO Cox regression. To prevent overfitting, 10-fold cross-validation over 1000 iterations was used to determine the optimal penalty parameter (λ), minimizing cross-validated partial likelihood deviance, and lambda.min was selected. A risk score (*UBTF*-PS) was calculated as: *UBTF*-PS = Σ (coefficient_i × expression_i). The median *UBTF*-PS value was employed to dichotomize patients into two risk strata: high and low. Outcome differences were quantified through KM survival curves in conjunction with log-rank statistical testing. The model’s discriminatory capacity was evaluated through time-dependent ROC curves, with AUC values computed at 1-, 3-, and 5-year follow-up milestones. DCA via the rmda R package [60] (v1.6) quantified net benefit for pre-dicting 1-, 3-, and 5-year survival, comparing four models: *UBTF*-PS alone, age alone, pTNM stage alone, and the combined model (*UBTF*-PS + Age + pTNM Stage), plus “treat all” and “treat none” strategies. External corroboration was conducted in the METABRIC cohort employing identical risk formula and cutoff values derived from the TCGA-BRCA cohort.

### 4.11. Construction of Nomogram

To appraise the independent prognostic utility of *UBTF*-PS, clinical variables including age, race (Asian vs. non-Asian), TNM stage (I-II vs. III-IV), and risk score (*UBTF*-PS) underwent preliminary univariate Cox regression analysis. Variables achieving *p* < 0.05 were then entered into multivariable Cox regression for pinpointing independent prognostic factors. An integrated nomogram featuring the identified independent prognostic factors was built to forecast individualized survival probability at 1-, 3-, and 5-year time points. Nomogram discriminatory capacity was quantified through time-dependent ROC curves, deriving AUC values. Calibration plots generated through the rms R package (v 6.2-0) served to evaluate the correspondence between model predictions and actual survival outcomes [61].

### 4.12. Cell Culture and CRISPR/Cas9-Mediated UBTF Knockdown

The BT-549 BRCA cell line originated from ATCC. LM2 was graciously supplied by Dr. Joan Massagué [62]. Cell propagation occurred in DMEM medium supplemented with 10% fetal bovine serum (FBS; Gibco) plus 1% penicillin-streptomycin under standard conditions (37 °C, humidified 5% CO_2_). Regular mycoplasma testing was conducted employing the MycoAlert Mycoplasma Detection Kit (Lonza, Shanghai, China).

To generate *UBTF* knockdown stable BRCA cell lines, we employed the CRISPR/Cas9 system. *UBTF*-targeting small-guide RNA (sgRNA) was engineered using the Optimized CRISPR Design program, which employs validated algorithms to maximize on-target activity and minimize off-target effects [63]. The sgRNA sequences are as follows: sg*UBTF*#1: 5′-TCTTCTCGGAGGAGAAACGG-3′; sg*UBTF*#2: 5′-CTCTGCAGTCCAAGTCGGTA-3′. 293T cells were employed to assemble and package Lenti-Cas9-sgRNA-puro expressing Cas9 nuclease coupled with *UBTF*-directed guide RNA, alongside the appropriate empty vector control. Lentivirus was introduced to cultured BRCA cells at a multiplicity of infection (MOI) of approximately 10. Following 72 h incubation, selection of stable cells was accomplished using puromycin at 2.0 μg/mL (Thermo Fisher, Beijing, China, A1113803) for 7 days. Pooled populations of puromycin-resistant cells were used for all subsequent experiments. Knockdown efficacy was corroborated via Western blot analysis in conjunction with qRT-PCR, demonstrating >70% reduction in *UBTF* protein and mRNA levels compared to control cells (Figure 6A).

### 4.13. RNA Isolation and qRT-PCR

Cells underwent total RNA extraction using TRIzol^®^ reagent (Invitrogen, Shanghai, China 15596026). For complementary DNA (cDNA) synthesis, 500 ng of the purified RNA was reverse-transcribed employing the Prime-Script™ RT Master Mix (Takara, Dalian, China, RR037A). qRT-PCR was subsequently executed employing the ChamQ Universal SYBR Green Master Mix (Vazyme, Nanjing, China, Q711). The amplification protocol entailed an initial denaturation phase at 95 °C for 10 min, with subsequent 40 cycles at 95 °C for 15 s and 60 °C for 60 s. Melting curve analysis was executed to corroborate amplicon specificity. Quantification of gene expression employed the 2^−ΔΔCT^ method, with normalization performed relative to GAPDH as the internal control. Primer sequences for this investigation are specified below: *UBTF* (Forward, 5′- AAACCACCGAATCACACATGG-3′; Reverse, 5′-TCTGTCAATGTACGGAACTTCCT-3′).

### 4.14. Western Blotting

Protein specimens were isolated from cultured cells through extraction in T-PER protein extraction reagent (Thermo Fisher) alongside protease and phosphatase inhibitor cocktail (Thermo Fisher), followed by incubation on ice for 30 min to ensure complete cell lysis. Upon centrifugation at 11,000× *g* for 20 min at 4 °C, supernatant fractions were isolated, and protein content was quantified via a BCA Protein Assay Kit (Thermo Fisher, 23225). For immunoblotting, protein samples (40 μg) were loaded and electrophoresed by SDS-PAGE employing 8–15% polyacrylamide gels under denaturing conditions. Proteins were then electrotransferred onto polyvinylidene fluoride (PVDF) membranes (Millipore, Burlington, MA, USA, IPVH00010). Succeeding transfer, blocking was performed using 5% non-fat milk prepared in Tris-buffered saline with 0.1% Tween-20 (TBST; Sangon Biotech, Shanghai, China, B040126), incubating membranes for 2 h at ambient temperature. Membranes were subsequently incubated with specific primary antibodies at 4 °C overnight while gently rocking. Primary antibodies employed were *GAPDH* (diluted 1:20,000, Proteintech, Wuhan, China, 60004-1-Ig), *UBTF* (diluted 1:500, Abcam, Shanghai, China, EP2741Y), Nucleolin (diluted 1:1000, Abcam, ab22758), *CD274/PD-L1* (diluted 1:1000, Cell Signaling Technology, Shanghai, China, #13684), *PDCD1LG2/PD-L2* (diluted 1:1000, Cell Signaling Technology, Shanghai, China, #82723), mTOR (diluted 1:1000, Cell Signaling Technology, #2983), *p-mTOR* (Ser2448) (diluted 1:1000, Cell Signaling Technology, #5536), *ERK1/2* (diluted 1:1000, Cell Signaling Technology, #4695), *p-ERK1/2* (Thr202/Tyr204) (diluted 1:1000, Cell Signaling Technology, #4370), *MEK1/2* (diluted 1:1000, Cell Signaling Technology, #9122), and *p-MEK1/2* (Ser217/221) (diluted 1:2000, Cell Signaling Technology, #9154). Following three washes in TBST (15 min each), HRP-conjugated goat anti-rabbit IgG secondary antibody (diluted 1:10,000, SAB, Shanghai, China, L3012) or HRP-conjugated goat anti-mouse IgG secondary antibody (diluted 1:10,000, SAB, L3032) was introduced with subsequent 2 h incubation at ambient temperature. Protein bands were revealed employing LumiBest ECL reagent solution kit (share-bio, Shanghai, China, WB012).

### 4.15. Cell Proliferation Assessment

Cellular proliferative capacity was dynamically appraised employing the CCK-8 (SAB biotech, Nanjing, China, CP002). Briefly, 2500 cells suspended in 100 μL complete medium were added per well to 96-well plates, with subsequent cultivation extending up to 7 days. At predefined intervals (days 1 through 6), wells were given 10 μL CCK-8 solution and kept for a further 2 h. Measurements at 450 nm were obtained employing a microplate reader (BioTek Synergy H1, Beijing, China) to appraise metabolic activity as a surrogate for viable cell number. Background optical density from media-only wells was deducted from all measurements.

### 4.16. Colony Formation Assay

Clonogenic capacity of cells was appraised via colony formation assay. Six-well plates received approximately 500 cells per well, which were then cultured for 14 days under standard conditions to permit colony maturation, with medium refreshed every 3 days. Following the emergence of visible, distinct monoclonal colonies, the growth medium was aspirated, and adherent cells were rinsed with PBS (Solarbio, Beijing, China) and then immobilized with 4% paraformaldehyde at ambient temperature for 45 min. Upon completion of fixation, colonies received 0.1% crystal violet staining for 15 min, followed by gentle distilled water washes to clear excess dye, then air-drying. Three independent investigators, blinded to experimental groups, manually counted colonies exceeding 50 cells under light microscopy. Clonogenic efficiency was computed as (colonies enumerated/cells initially plated) × 100%, and outcomes were normalized to the control group.

### 4.17. Cellular Migratory Capacity Assessment via Transwell

To appraise cellular migratory capabilities, Transwell assays were executed employing Transwell Permeable Supports (Corning Costar, Beijing, China, #3422) in 24-well plates. Cells were harvested by trypsinization, enumerated employing an automated cell counter, and re-suspended in serum-free DMEM medium. To exclude the confounding influence of proliferation, mitomycin C (10 μg/mL, Sigma-Aldrich, Shanghai, China, M4287) was applied to cells for 2 h as pretreatment. Thereafter, 5000 viable cells resuspended in 200 μL serum-free DMEM with mitomycin C (10 μg/mL) were transferred to the upper chamber; concurrently, 600 μL complete DMEM medium enriched with 20% FBS was dispensed into the lower chamber to serve as chemoattractant. After 18 h at 37 °C in 5% CO_2_, non-migrated cells were removed with a cotton swab. Cells on the lower membrane surface were fixed in 4% paraformaldehyde (15 min), stained with 0.5% crystal violet (20 min), and photographed at 100× magnification. Five random fields per insert were analyzed for migrated cell counts using ImageJ software (v2.0.0). Fold-change values are normalized to the control group.

### 4.18. Cellular Motility Assessment via Scratch Wound

Cell motility was further assessed by wound healing assay. Cells were seeded at 5 × 10^5^ cells/well in 6-well plates and cultured to 80–90% confluence (~24 h). To exclude proliferation, cells were pretreated with mitomycin C (10 μg/mL) for 2 h before wounding. Using a sterile 200 μL pipette tip positioned perpendicular to the plate, a linear scratch was made across the cell monolayer. Two PBS washes removed detached cells and debris, and fresh complete DMEM medium containing mitomycin C (10 μg/mL) was supplemented. Cellular migration was appraised by quantifying the movement of cells into the acellular area generated by the scratch. Wound closure was monitored and photographed under phase-contrast microscopy immediately (0 h) and 12 h after scratching employing an inverted microscope (Nikon Eclipse Ti-U, Shanghai, China). Images were captured at 100× magnification from at least three fields per well. Wound width was quantified employing ImageJ software.

### 4.19. Apoptotic Cell Death Assessment via Flow Cytometry

Apoptotic cell death was appraised employing the Annexin V-FITC and PI Apoptosis Detection Kit (BD Biosciences, Beijing, China, 556547) in conjunction with flow cytometric analysis. Cells were maintained to approximately 70–80% confluence, then both floating and adherent cells were harvested by trypsinization (for adherent cells) and gentle pipetting (for floating cells), combined, centrifuged (400× *g*, 5 min), and rinsed (ice-cold PBS) to eliminate residual medium and debris. Cell pellets underwent resuspension in 1× binding buffer to achieve 1 × 10^7^ cells/mL. Aliquots of 100 μL were dispensed into 1.5 mL tubes and labeled with 5 μL Annexin V-FITC plus 5 μL PI as per manufacturer’s guidelines. Following incubation in the dark at ambient temperature for 15 min, 400 μL of 1× binding buffer was supplemented to each tube. Specimens were immediately interrogated by flow cytometry (BD FACSCanto II, Beijing, China) within 1 h. For each specimen, at least 10,000 events were recorded. Cell populations were quantified employing FlowJo software (v10.8.1).

### 4.20. Statistical Analysis

Each in vitro functional experiment was repeated three times in three independent experimental replicates. Statistical analyses were executed employing R software (v4.3.2). Comparisons between two groups utilized the Wilcoxon rank-sum test (Mann–Whitney U test), and comparisons across multiple groups utilized the Kruskal–Wallis test with post hoc pairwise comparisons and multiple-testing correction as appropriate. For in vitro functional experiments, appropriate parametric or non-parametric statistical tests were applied as specified in figure legends. Spearman’s rank correlation coefficient was employed for correlation and association analyses between continuous variables. When comparing *UBTF* high- versus low-expression groups for differential gene expression, multiple testing correction was applied to raw *p* values via the Benjamini–Hochberg FDR method; genes were designated as DEGs if they satisfied both FDR-adjusted *p* value < 0.05 and |log_2_ FC| > 1. Significance levels were designated as follows: *p* < 0.05, *p* < 0.01, and *p* < 0.001.

## 5. Conclusions

*UBTF* serves as a promising biomarker for prognostic prediction and immunotherapy response across multiple cancers, particularly BRCA. *UBTF* expression correlates with immune microenvironment composition, immune checkpoint molecule regulation, and patient survival outcomes. Functional validation in BRCA demonstrates that *UBTF* promotes tumor cell proliferation and migration through *mTOR/ERK/MEK* signaling activation while suppressing *PD-L1* and *PD-L2* expression. These findings warrant further investigation of *UBTF* as a therapeutic target for cancer treatment and immunotherapy optimization.

## Data Availability

The original contributions presented in this study are included in the Supplementary Material. Further inquiries can be directed to the corresponding authors.

## Supplementary Materials

The following supporting information can be downloaded at: https://www.mdpi.com/article/10.3390/ijms27062909/s1.
