# Supplementary material for: Pancancer Analysis and the Oncogenic Role of UBTF in Breast Invasive Carcinoma
Source: Int J Mol Sci. 2026 Mar 23;27(6):2909. doi: 10.3390/ijms27062909 (PMC13027293; doi:10.3390/ijms27062909)
Supplement: Supplementary file 1 [file ijms-27-02909-s001.zip › ijms-4156983-supplementary.pdf]

Pancancer analysis and the oncogenic role of UBTF in Breast Invasive Carcinoma

Supplementary Table

Supplementary Table S1. 33 kinds of cancer detailed names and sample information

Type	Description	Tumor samples (TCGA)	Normal samples (TCGA+GTEx)
ACC	Adrenocortical carcinoma	79	258
BLCA	Bladder Urothelial Carcinoma	406	40
BRCA	Breast invasive carcinoma	1101	572
CESC	Cervical squamous cell carcinoma and endocervical adenocarcinoma	306	22
CHOL	Cholangiocarcinoma	35	9
COAD	Colon adenocarcinoma	455	820
DLBC	Lymphoid Neoplasm Diffuse Large B-cell Lymphoma	48	929
ESCA	Esophageal carcinoma	163	1456
GBM	Glioblastoma multiforme	153	2647
HNSC	Head and Neck squamous cell carcinoma	504	44
KICH	Kidney Chromophobe	65	114
KIRC	Kidney renal clear cell carcinoma	532	161
KIRP	Kidney renal papillary cell carcinoma	290	121
LAML	Acute Myeloid Leukemia	150	0
LGG	Brain Lower Grade Glioma	513	2642
LIHC	Liver hepatocellular carcinoma	371	276
LUAD	Lung adenocarcinoma	516	637
LUSC	Lung squamous cell carcinoma	501	627
MESO	Mesothelioma	87	0
OV	Ovarian serous cystadenocarcinoma	376	180
PAAD	Pancreatic adenocarcinoma	179	332
PCPG	Pheochromocytoma and Paraganglioma	181	3
PRAD	Prostate adenocarcinoma	498	297
READ	Rectum adenocarcinoma	165	789
SARC	Sarcoma	260	2
SKCM	Skin Cutaneous Melanoma	471	1810
STAD	Stomach adenocarcinoma	375	391
TGCT	Testicular Germ Cell Tumors	134	361
THCA	Thyroid carcinoma	512	712
THYM	Thymoma	120	2
UCEC	Uterine Corpus Endometrial Carcinoma	545	177
UCS	Uterine Carcinosarcoma	57	142
UVM	Uveal Melanoma	80	0

Supplementary Figures

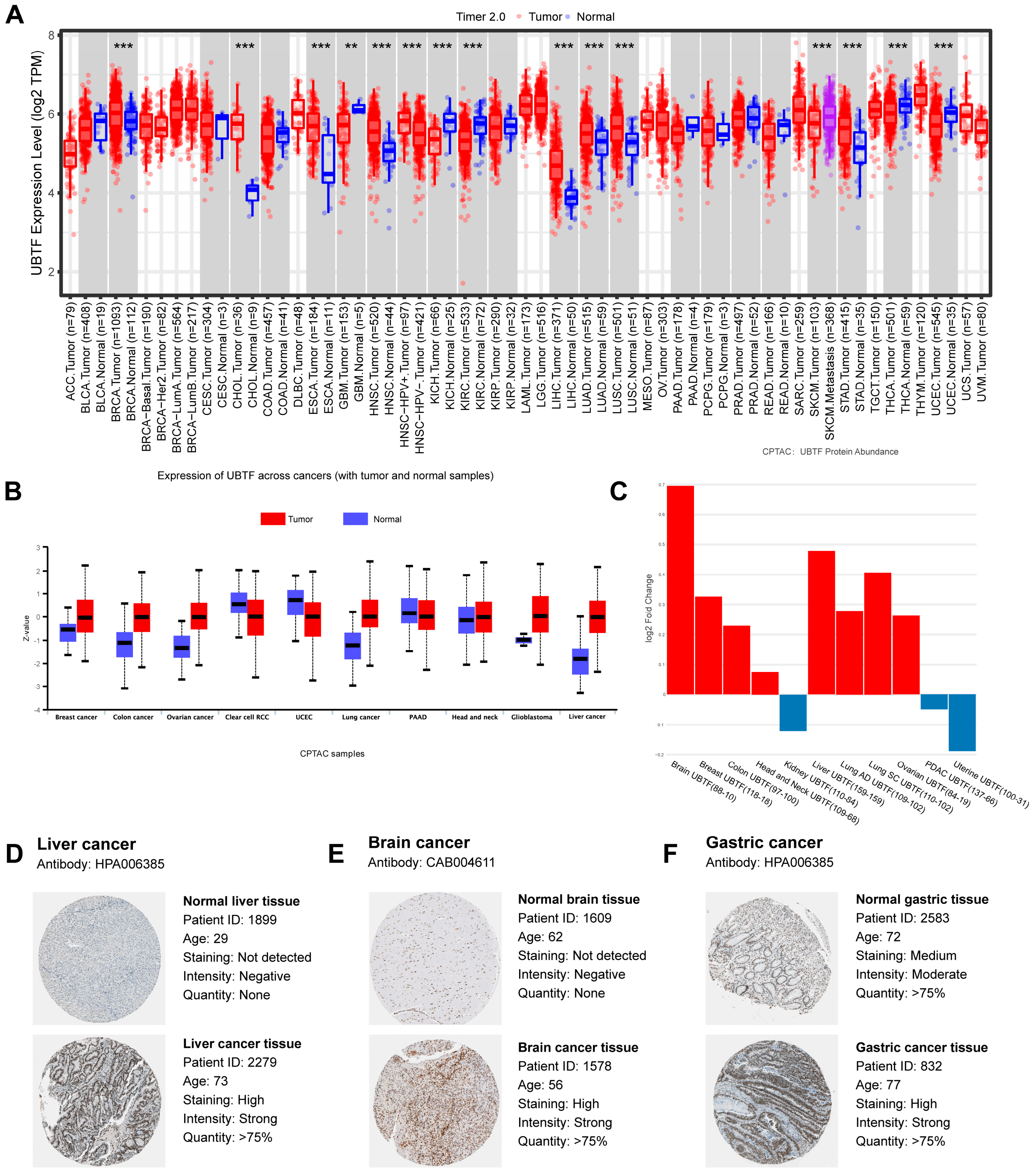

Supplementary Figure S1. (**A**) The mRNA expression of UBTF from the TIMER database; (**B**) The mRNA expression of UBTF from the CPTAC database; (**C**) The protein expression of UBTF from the CPTAC database; (**D-F**) HPA dataset validates the protein expression of UBTF in various cancers. **, P < 0.01; ***, P < 0.001.

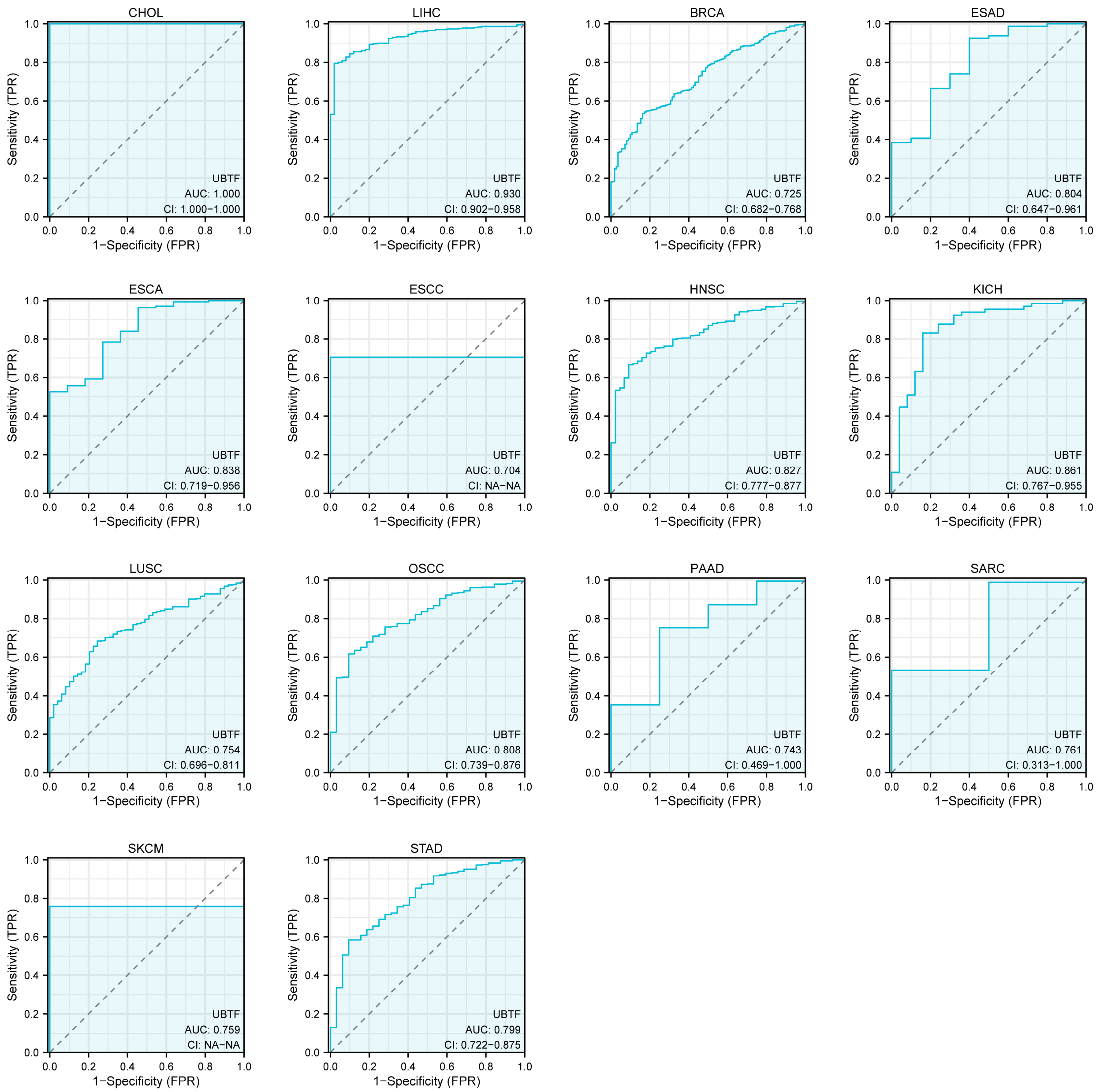

Supplementary Figure S2. The tumors with the AUC values greater than 0.7.

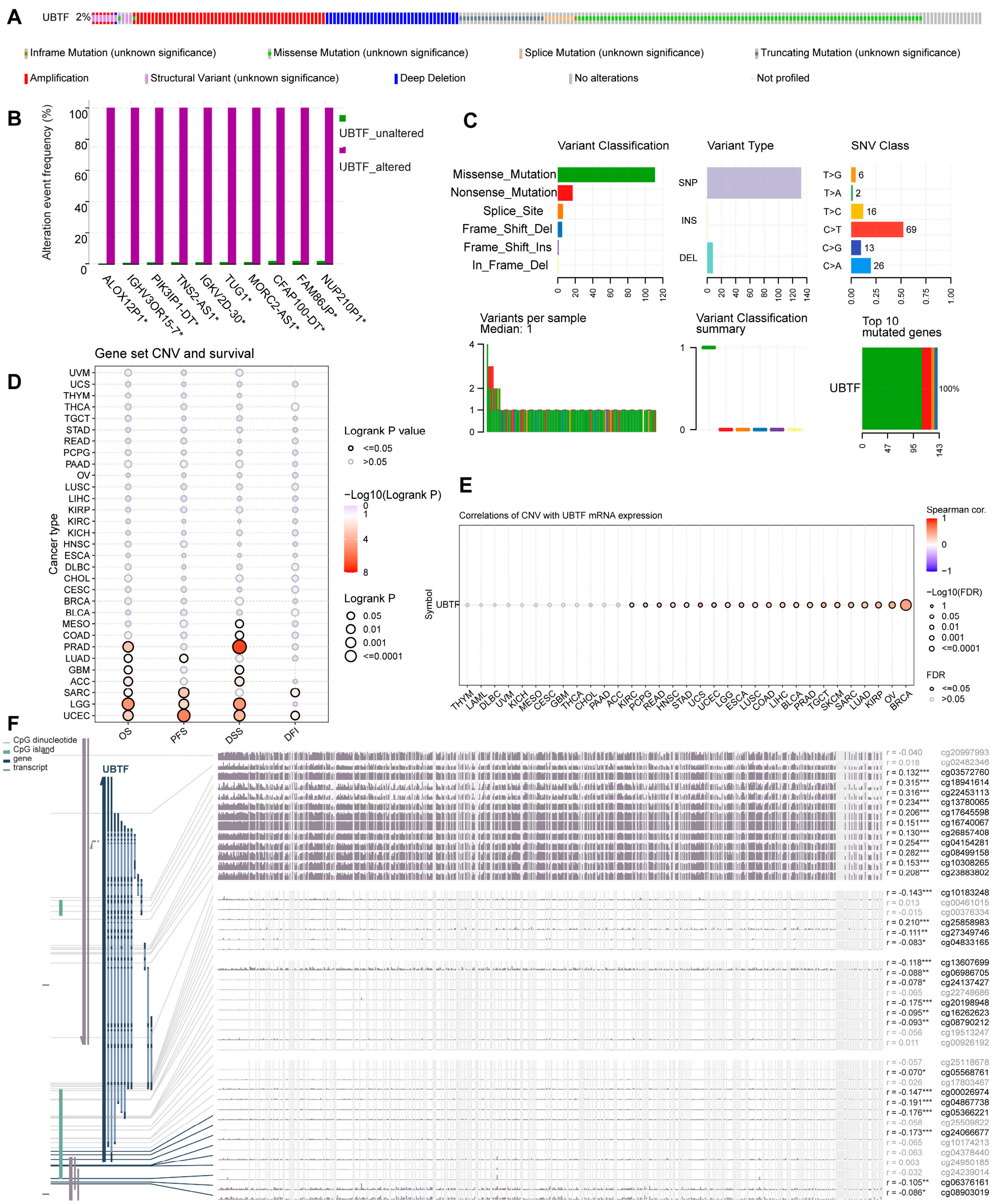

Supplementary Figure S3. (A) The mutation frequency of UBTF in pan-cancer patients; **(B)** TOP 10 differential genes between UBTF altered group and UBTF unaltered group; **(C)** Summaries of UBTF SNP in pan-cancer; **(D)** The correlation between UBTF CNV and survival; **(E)** The correlation between CNV and UBTF expression; **(F)** Schematic map of the human UBTF gene including the relative positions and CpG sites based on 45 methylation probes in BRCA within a predicted CpG island. Correlation between mRNA expression and methylation of UBTF.

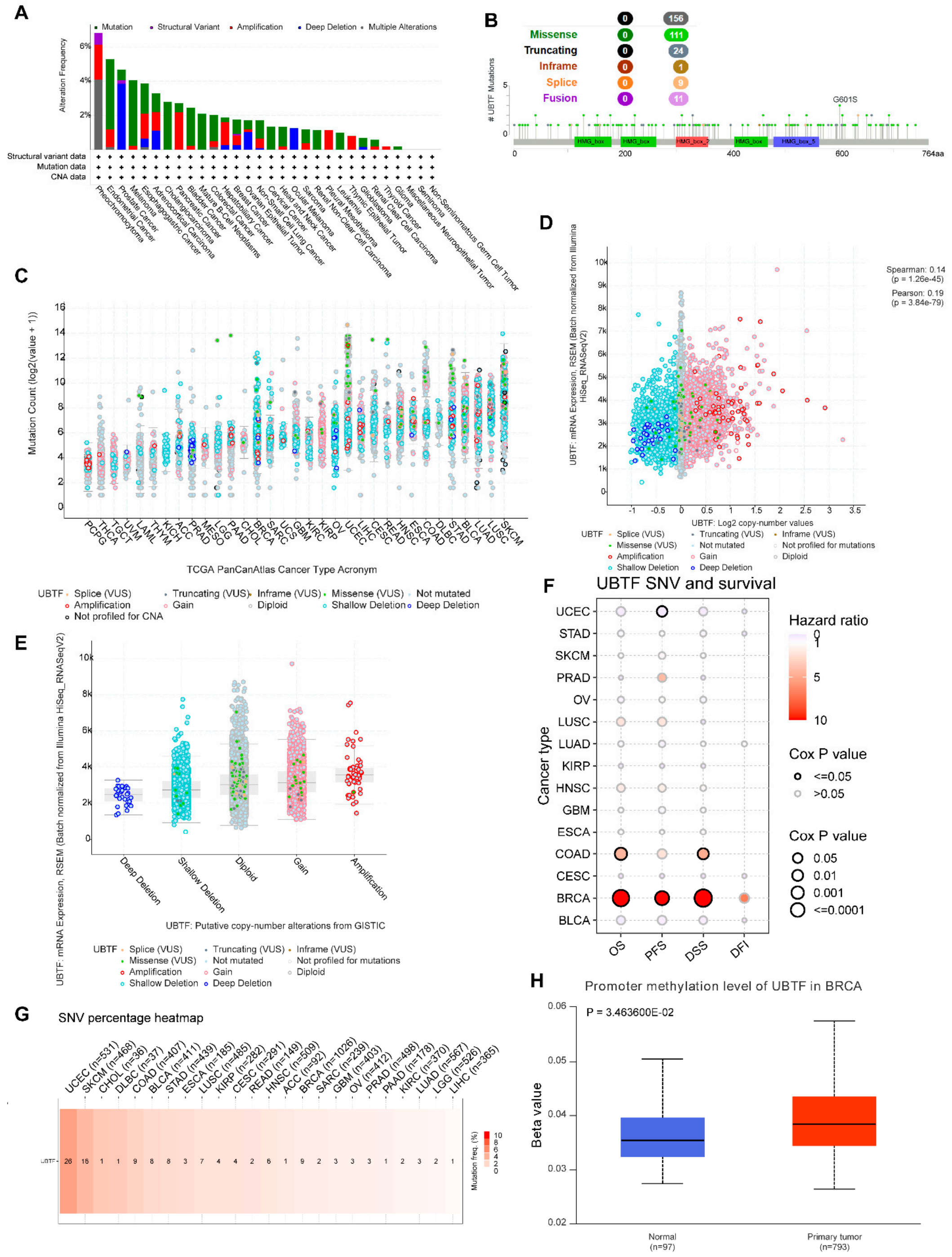

Supplementary Figure S4. (**A**) Genetic alteration frequency of UBTF. (**B**) UBTF mutation sites. (**C**) UBTF mutation counts. (**D**) Correlations between UBTF expression and copy number values in the pancancer pa-tients. (**E**) Differences in UBTF expression among different copy number alterations. (**F**) Correla-tions between UBTF SNVs and survival in patients with various cancers. (**G**) The profile of SNVs of UBTF in various cancers. (**H**) The correlation between the methylation and mRNA expression of UBTF in BRCA.

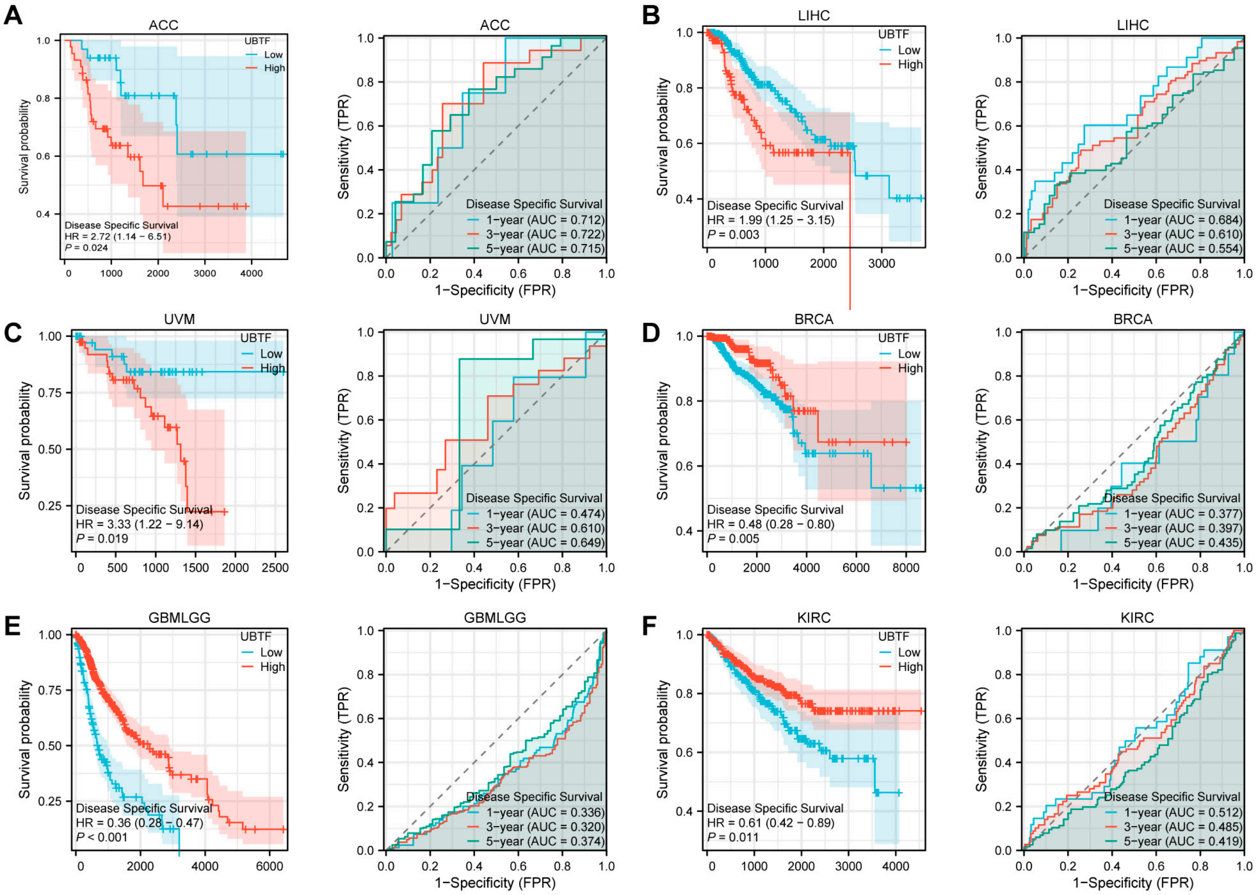

Supplementary Figure S5. Kaplan–Meier survival curves and ROC analysis between UBTF expression and DSS.

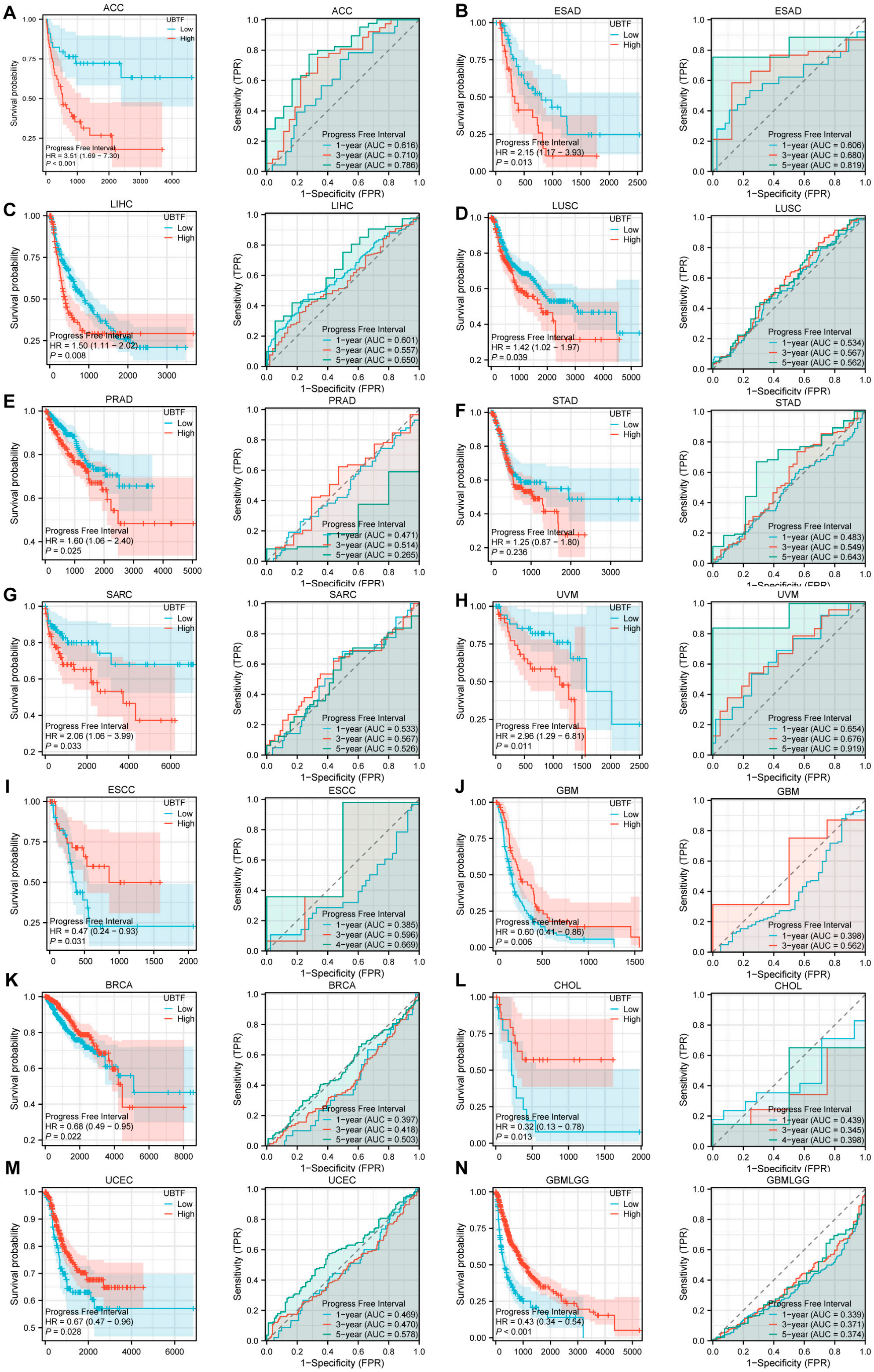

Supplementary Figure S6. Kaplan–Meier survival curves and ROC analysis between UBTF expression and PFI.

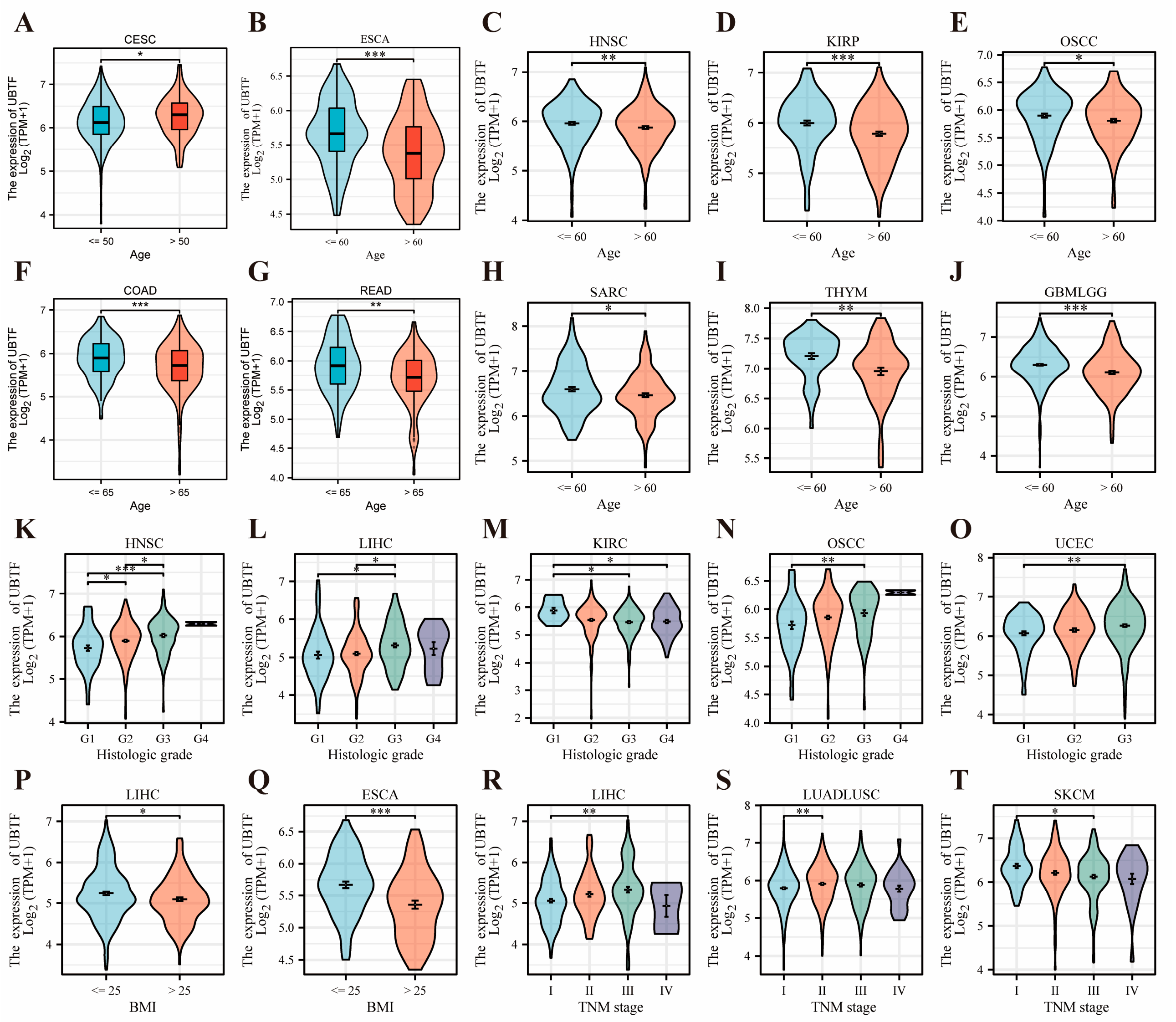

Supplementary Figure S7. The correlation between UBTF and clinical parameters. (**A-J**) Differences of UBTF ex-pression between age ≤ 50 and age > 50. (**K-O**) Differences of UBTF expression among various tumor grades. (**P-Q**) Differences of UBTF expression among various BMI. (R-T) Differences of UBTF expression among various TNM stages. * P < 0.05; ** P < 0.01; *** P < 0.001.

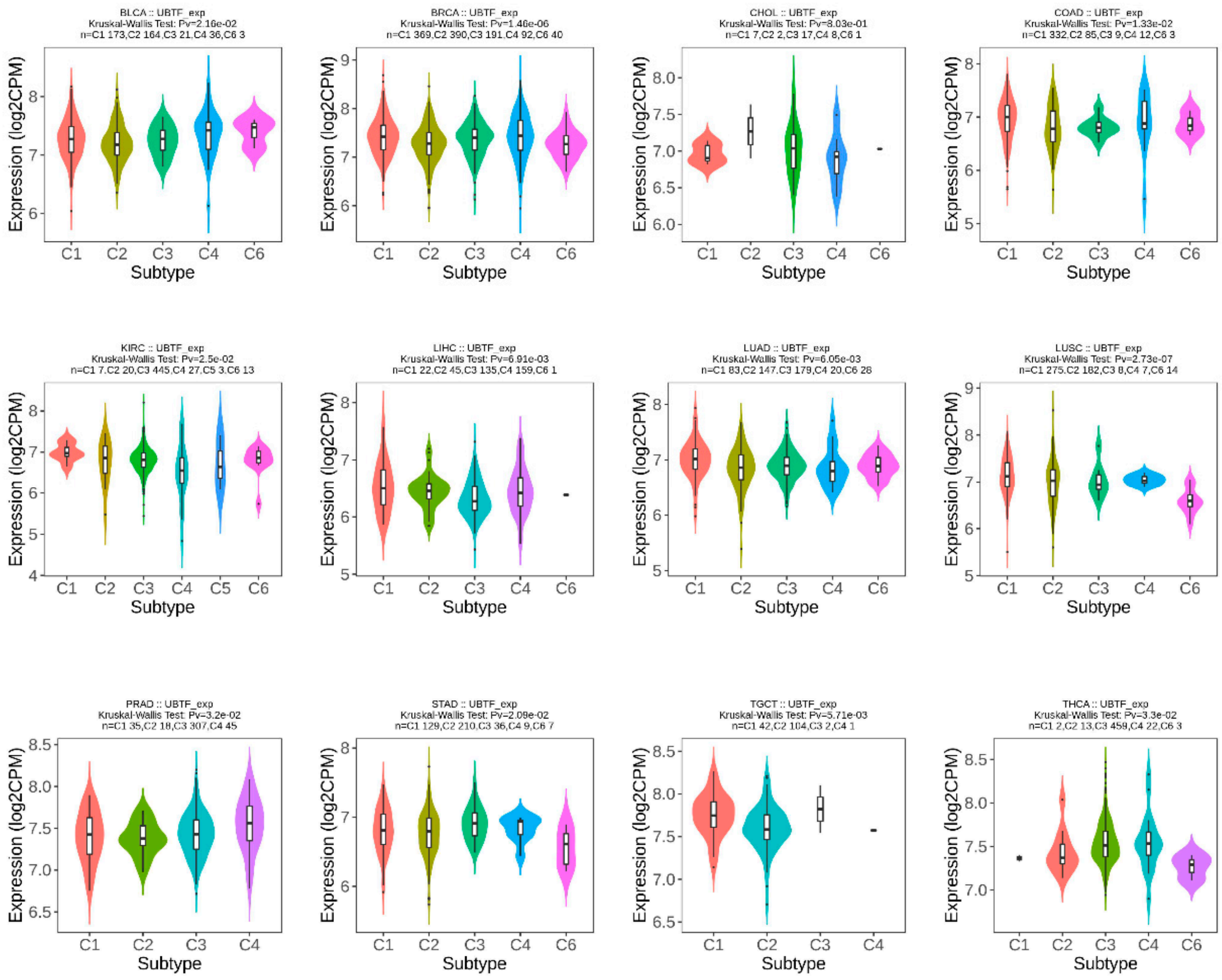

Supplementary Figure S8. Differences of UBTF expression among various immune subtypes, including C1 to C6.

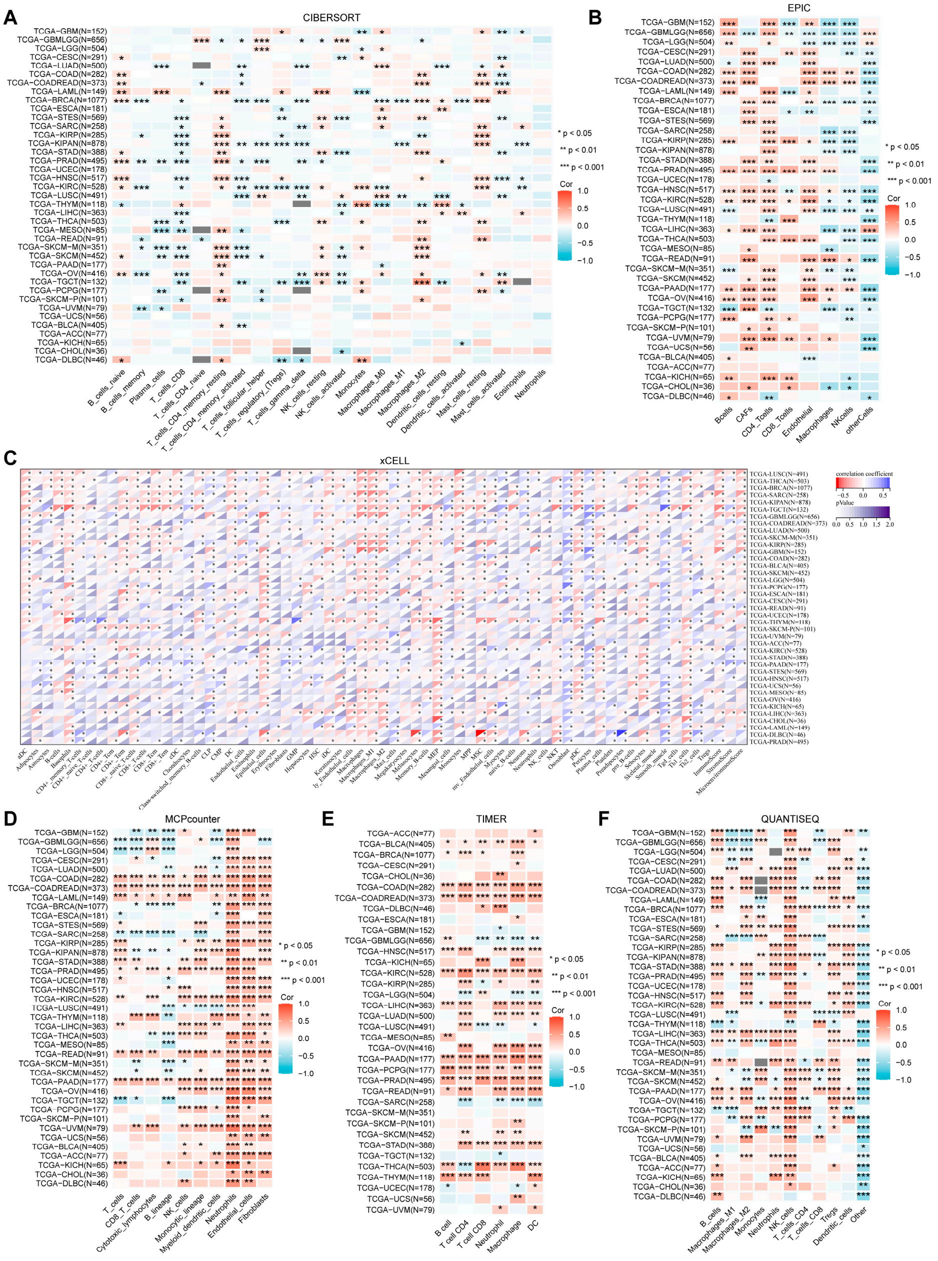

Supplementary Figure S9. (A) Pan-cancer analysis of the correlation between C15orf48 and immune cell infiltration (CIBERSORT); **(B)** Pan-cancer analysis of the correlation between C15orf48 and immune cell infiltration (EPIC); **(C)** Pan-cancer analysis of the correlation between C15orf48 and immune cell infiltration (xCELL); **(D)** Pan-cancer analysis of the correlation between C15orf48 and immune cell infiltration (MCP-counter); **(E)** Pan-cancer analysis of the correlation between C15orf48 and immune cell infiltration (TIMER); **(F)** Pan-cancer analysis of the correlation between C15orf48 and immune cell infiltration (QUANTISEQ).

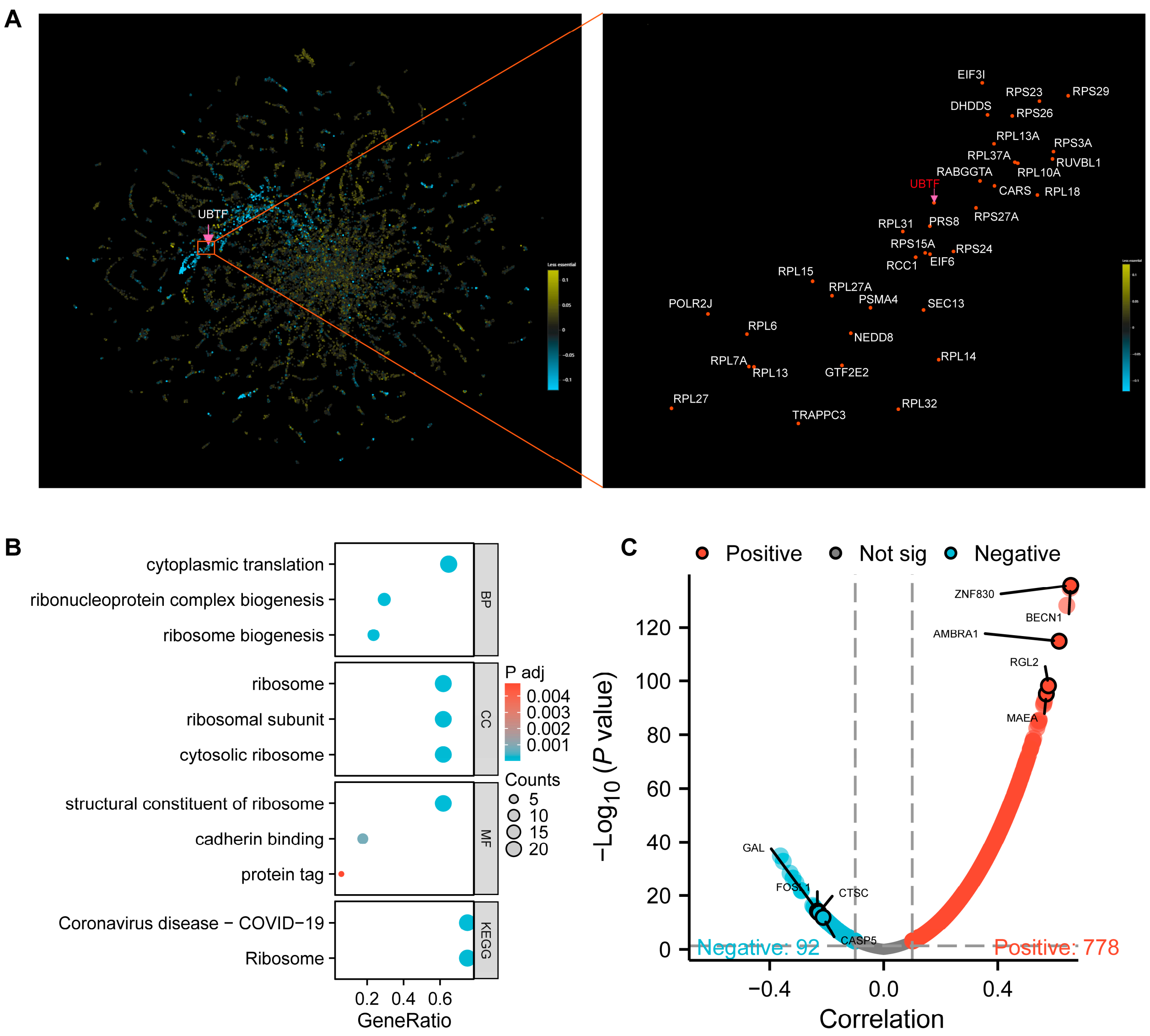

Supplementary Figure S10. (**A**) Co-expression analysis network of UBTF in BRCA; (**B**) Functional enrichment analysis of UBTF neighbor genes; (**C**) Correlation analysis between UBTF and apoptosis-related genes.

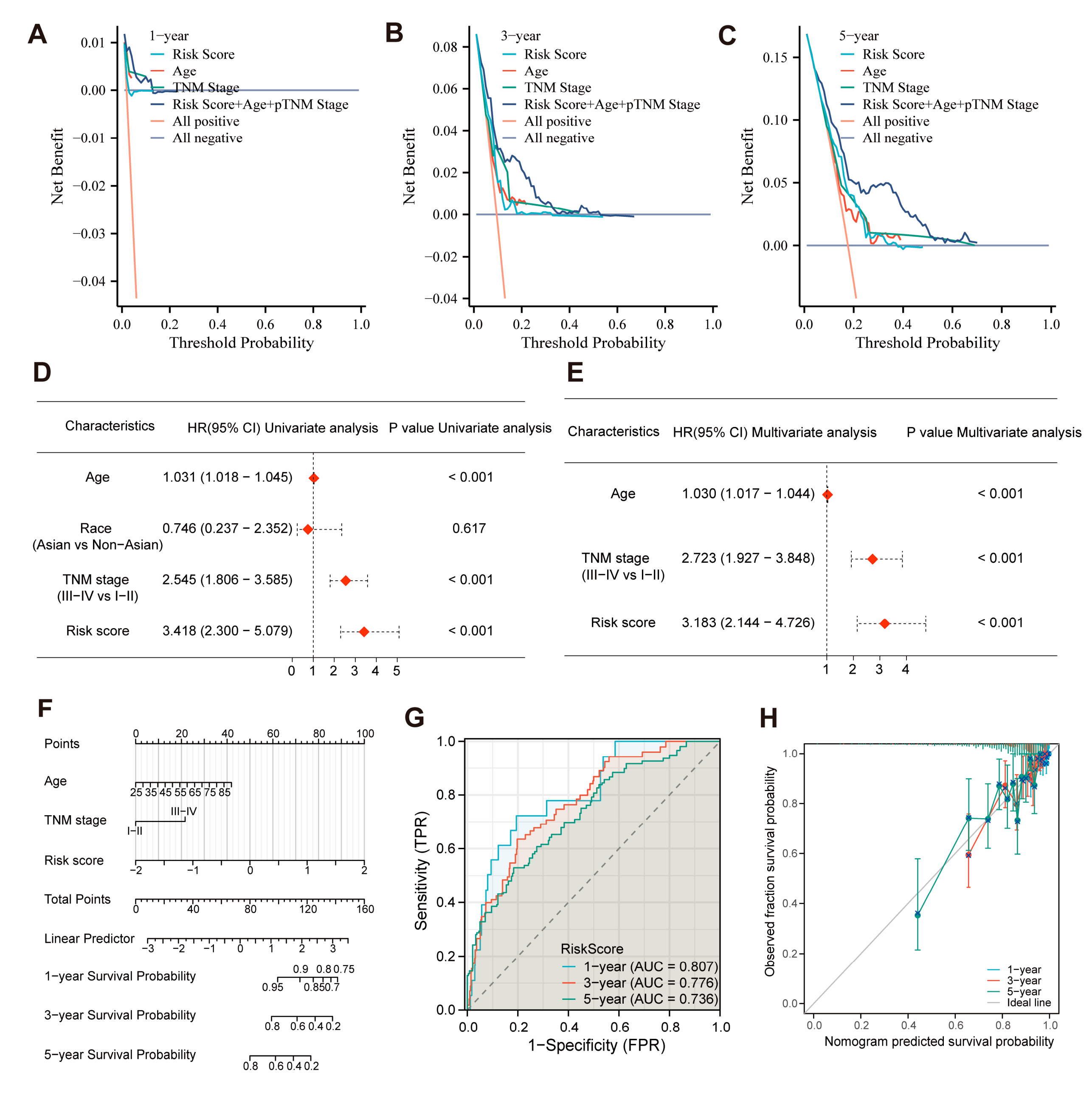

Supplementary Figure S11. Decision Curve Analysis (DCA) comparing the clinical utility of UBTF-PS model with standard clinical models for predicting 1-year (**A**), 3-year (**B**), and 5-year (**C**) survival in BRCA patients. Univariate (**D**) and multivariate (**E**) Cox analyses of selected variables in BRCA; (**F**) Nomogram based on independent risk factor in BRCA; (**G, H**) Time-dependent ROC analysis and calibration curve of the nomogram.

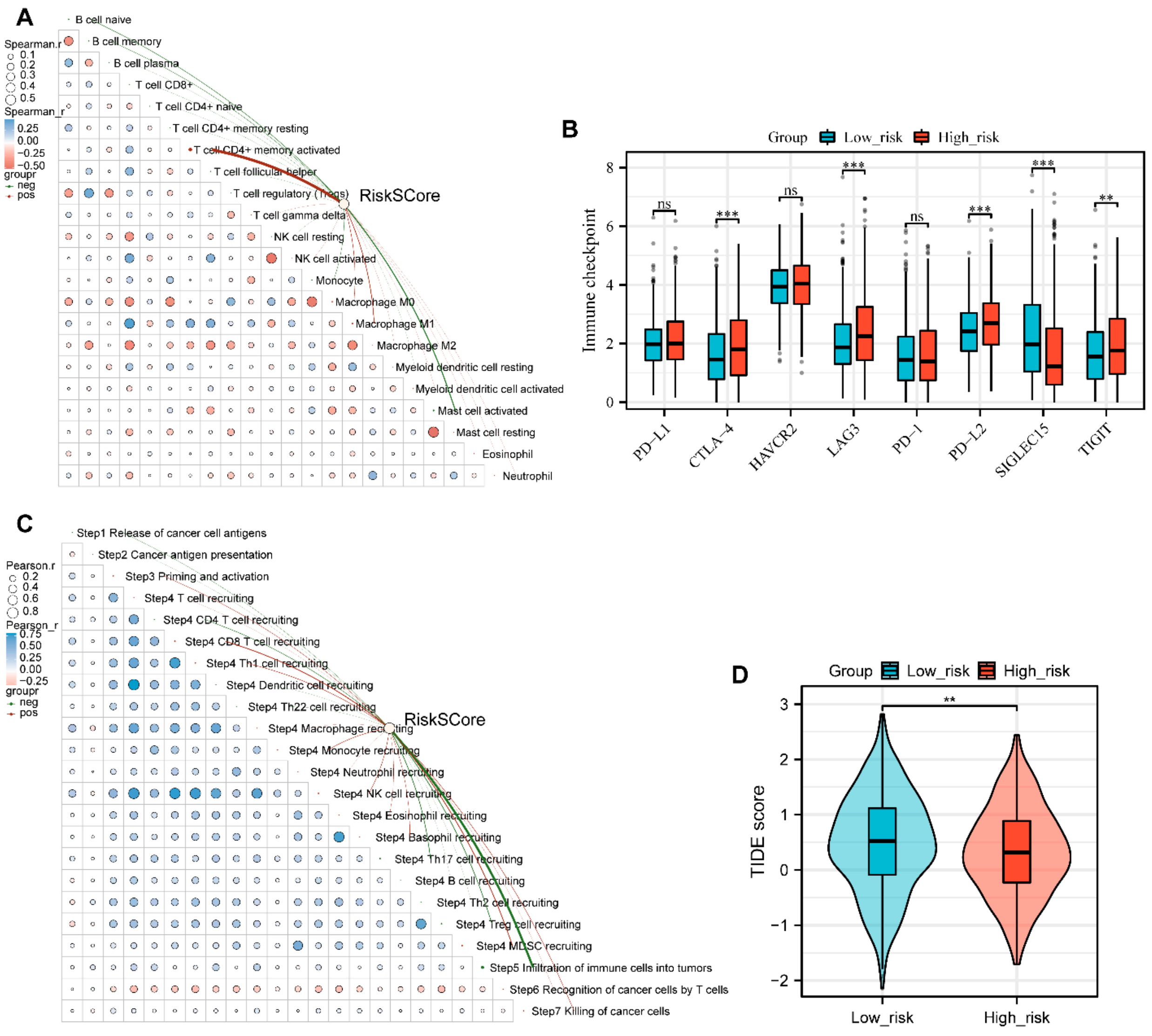

Supplementary Figure S12. (A) Correlation between the risk score and immune cell infiltration in BRCA; **(B)** Correlation between the risk score and immune checkpoint genes in BRCA; **(C)** Correlation between the risk score and immune steps in BRCA; **(D)** Correlation between the risk score and TIDE score in BRCA.

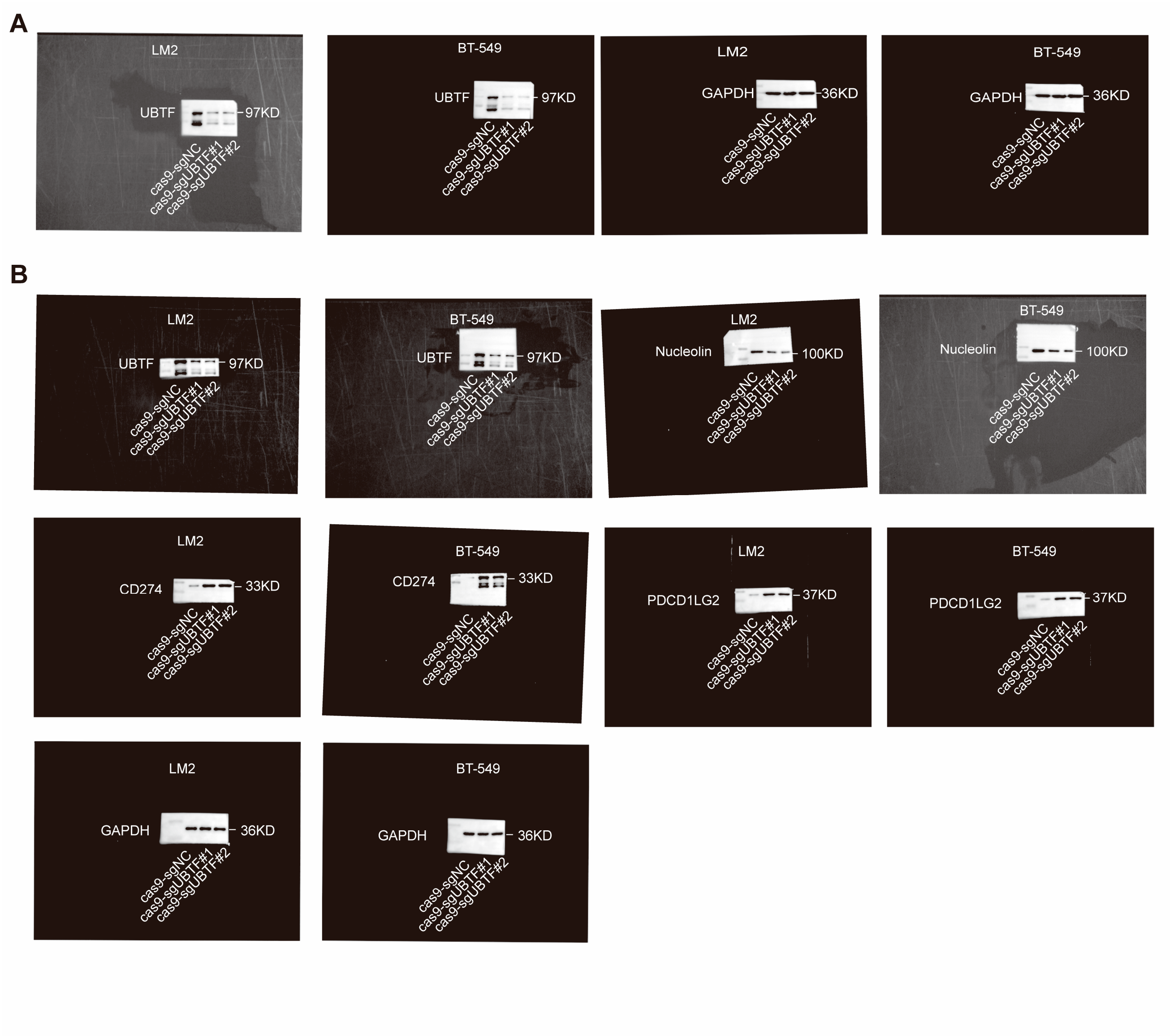

Supplementary Figure S13. (A, B) Original blots for data presented in Figure 6A and Figure 6H. (Top panels). (Bottom panels) Immunoblotting of GAPDH served as the loading control. The full-length gels are displayed with molecular weight markers (kDa) indicated. All samples within the same cell line were run on the same gel for comparative analysis. Background noise is retained to reflect the original imaging conditions.

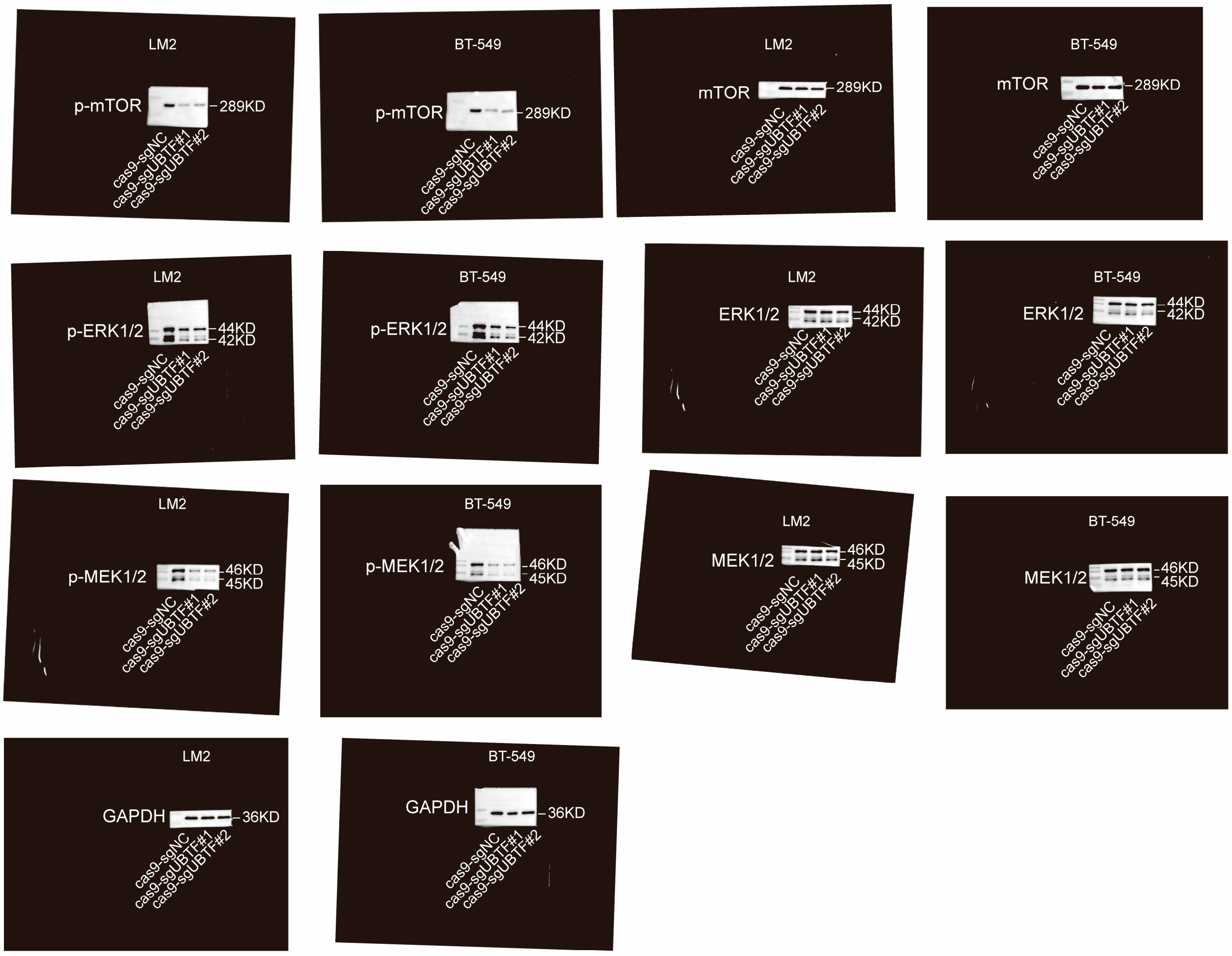

Supplementary Figure S14. Original blots for data presented in Figure 6I. (Bottom panels) Immunoblotting of GAPDH served as the loading control. The full-length gels are displayed with molecular weight markers (kDa) indicated. All samples within the same cell line were run on the same gel for comparative analysis. Background noise is retained to reflect the original imaging conditions.
